# What is in the Dust? Species‐Level Profiling Reveals Pathogenic and Protective Potential of Microbial Communities in Daycare and Home Environments

**DOI:** 10.1111/1758-2229.70433

**Published:** 2026-10-14

**Authors:** Jill A. McKay, Richard Harding‐Crooks, Vartul Sangal

**Affiliations:** ^1^ School of Geography and Natural Sciences, Faculty of Science and Environment Northumbria University Newcastle upon Tyne UK

**Keywords:** cancer, childhood leukaemia, daycare, dust microbiome, home biome, infectious diseases

## Abstract

Indoor environments are rich in microbial diversity, which is influenced by multiple factors, including human activities, occupancy levels and the presence of pets. Environment‐associated taxa were found to be prevalent in daycare and human‐associated taxa in home environments during the COVID‐19 pandemic, a period marked by profoundly altered social mixing and hygiene practices. In this study, we investigated the structure of microbial communities and their ecological profiles in daycare and home dust in the post‐COVID‐19 era. Microbial communities in household dust remain more heterogeneous, with a higher prevalence of human‐associated species, than daycare samples. Full‐length 16S rRNA gene sequencing enabled species‐level taxonomic resolution, yet approximately 40% of amplicon sequence variants remained unclassified, underscoring the vast, novel microbial diversity within our indoor environments encountered in everyday life. AI‐assisted ecological profiling revealed a higher burden of pathogens and opportunistic pathogens in home environments than in daycares. Interestingly, the presence of pets had limited influence on microbial diversity in household dust. Finally, several species with known probiotic properties and immunostimulatory potential were present in both environments, reinforcing the notion that microbial exposure during the critical window of early childhood may confer protection against various non‐communicable diseases including asthma, childhood leukaemia and other cancers.

## Graphical abstract

Daycare and home dust predominantly harbour commensal and environmental bacteria; however, a significant proportion remains unclassified, indicating routine childhood exposure to novel microorganisms. Both environments contain species with probiotic and immunostimulatory properties that may confer protection against non‐communicable diseases including asthma, childhood leukaemia and other cancers early in childhood.

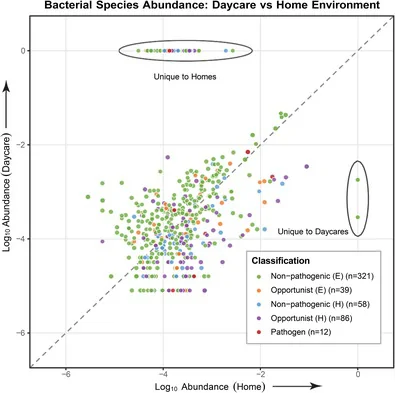

## 1 Introduction

Indoor environments harbour diverse microorganisms with members of phyla *Actinobacteria*, *Bacteroidetes*, *Firmicutes and Proteobacteria* (now *Actinomycetota*, *Bacteroidota*, *Bacillota* and *Pseudomonadota*, respectively) being most abundant (Park et al. 2021; Nygaard and Charnock 2018; McKay et al. 2024). Environmental taxa were predominant in daycares and school environments, whereas human‐associated bacteria were more abundant in dust samples from homes (Park et al. 2021; McKay et al. 2024). Microbial diversity is shaped by several factors, including human activities, temperature, humidity, occupancy levels, presence of animals/pets and plants (Tan et al. 2025; Guo et al. 2026; Chawla et al. 2023). For instance, a change in occupancy from adults to pre‐pubescent children in indoor environments was associated with a significant decrease in the abundance of *Propionibacterium* (now *Cutibacterium*), which is predominant in sebaceous‐rich areas of skin in adolescents and adults (Nygaard and Charnock 2018). While *Moraxella osloensis* and *Acinetobacter johnsonii* were predominant species in kindergartens in Qingdao, China, human‐associated taxa, including *Cutibacterium. acnes* and *Streptococcus* spp., were more common on hand‐touched objects such as picture books and toys (Lam et al. 2022). Environment‐associated taxa, *Paracoccus* spp. and *Pseudomonas* spp., were dominant in the samples collected from floors (Lam et al. 2022).

Humans are the most important contributors to microbial diversity in samples collected from home environments (McKay et al. 2024; Jarma et al. 2024; Thompson et al. 2021). Human‐associated genera, including *Staphylococcus*, *Streptococcus*, *Corynebacterium* and *Lactobacillus*, accounted for ~19% of all bacterial taxa detected in home settings (Jarma et al. 2024). In our previous study, we found human‐associated *Bacillus*, *Corynebacterium*, *Staphylococcus* and *Streptococcus* were more prevalent in household dust, whereas environmental taxa including *Aliterella*, *Deinococcus* and *Nakamurella* were more abundant in daycare samples during the COVID‐19 pandemic (McKay et al. 2024). Home dust harboured more diverse bacterial taxa than dust from daycare settings. Various taxa in both environments were reported to induce anti‐inflammatory and immunomodulatory responses with the potential to protect children from various non‐communicable diseases (NCDs) and infectious diseases (McKay et al. 2024). It is well known that an exposure to diverse microbes early in childhood is associated with a reduced risk of some NCDs such as development of asthma, leukaemia and other childhood cancers (Ajrouche et al. 2015; Bach and Chatenoud 2012; Greaves 2018; Lupo et al. 2014; Marcotte et al. 2014; Peppas et al. 2023; Pombo‐de‐Oliveira et al. 2023; Rudant et al. 2015). However, indoor environments may also contain pathogens that could be transmitted to occupants (Guo et al. 2026; Chawla et al. 2023; Jarma et al. 2024). Genes associated with antimicrobial resistance to different classes of antibiotics were identified in dust samples from homes and workplaces, indicating that indoor environments can be a source of antimicrobial‐resistant pathogens (Klvanova et al. 2024; Pearce et al. 2025). Exposure to some microbes (bacteria and fungi), combined with certain environmental factors such as poor air quality and smoking, has been linked to chronic lung diseases (Wolf and Lai 2020; Hulin et al. 2012). Most of these findings are based on identification of the microbiome up to the genus level, which can sometimes be misleading. For example, *Corynebacterium* is highlighted as a human‐associated taxon, but multiple species are also isolated from diverse marine and terrestrial environments (Dover et al. 2021). Some corynebacterial species are significant pathogens, such as *Corynebacterium diphtheriae* and *Corynebacterium ulcerans* (Burkovski and Sangal 2024; Sangal and Burkovski 2020), but many of them are commensal (Herman et al. 2025). However, some commensals are emerging as important opportunistic pathogens, for example, *Corynebacterium striatum* (Sangal et al. 2024). In order to identify the prevalence of commensal, pathogenic and environmental bacteria, we have applied full‐length 16S rRNA gene amplicon sequencing to dust samples collected from home and daycare environments. Unprecedented shifts in hygiene behaviour and social mixing during the COVID‐19 pandemic may have fundamentally altered indoor microbial ecosystems. Therefore, by characterising microbial diversity in daycare and home dust in the post‐pandemic period, this study offers critical insight into any shifts in indoor microbial community structures.

## 2 Results

### 2.1 Microbial Diversity in Dust Samples From Homes and Daycare Providers

Home dust samples harboured more diverse microorganisms in comparison to daycares. Out of 3709 normalised and filtered amplicon sequence variants (ASVs), 584 were unique to home dust (absent in daycare samples) while only 23 were unique to dust from daycare providers. Microbial richness and evenness were higher in dust samples collected from daycare providers than those collected from homes; however, the microbiome in the latter has been more heterogeneous (α‐diversity: Shannon *p* = 0.0039 and Simpson *p* = 0.0039; Figure 1A,B). Consistent with our previous observations (McKay et al. 2024), the microbial composition of both environments was distinct from each other with some overlap, confirming that some microbial taxa were shared between the two environments (Figure 1C; PERMANOVA *p* = 0.001). However, the prevalence of some of these taxa between the two environments was significantly different.

**FIGURE 1:**
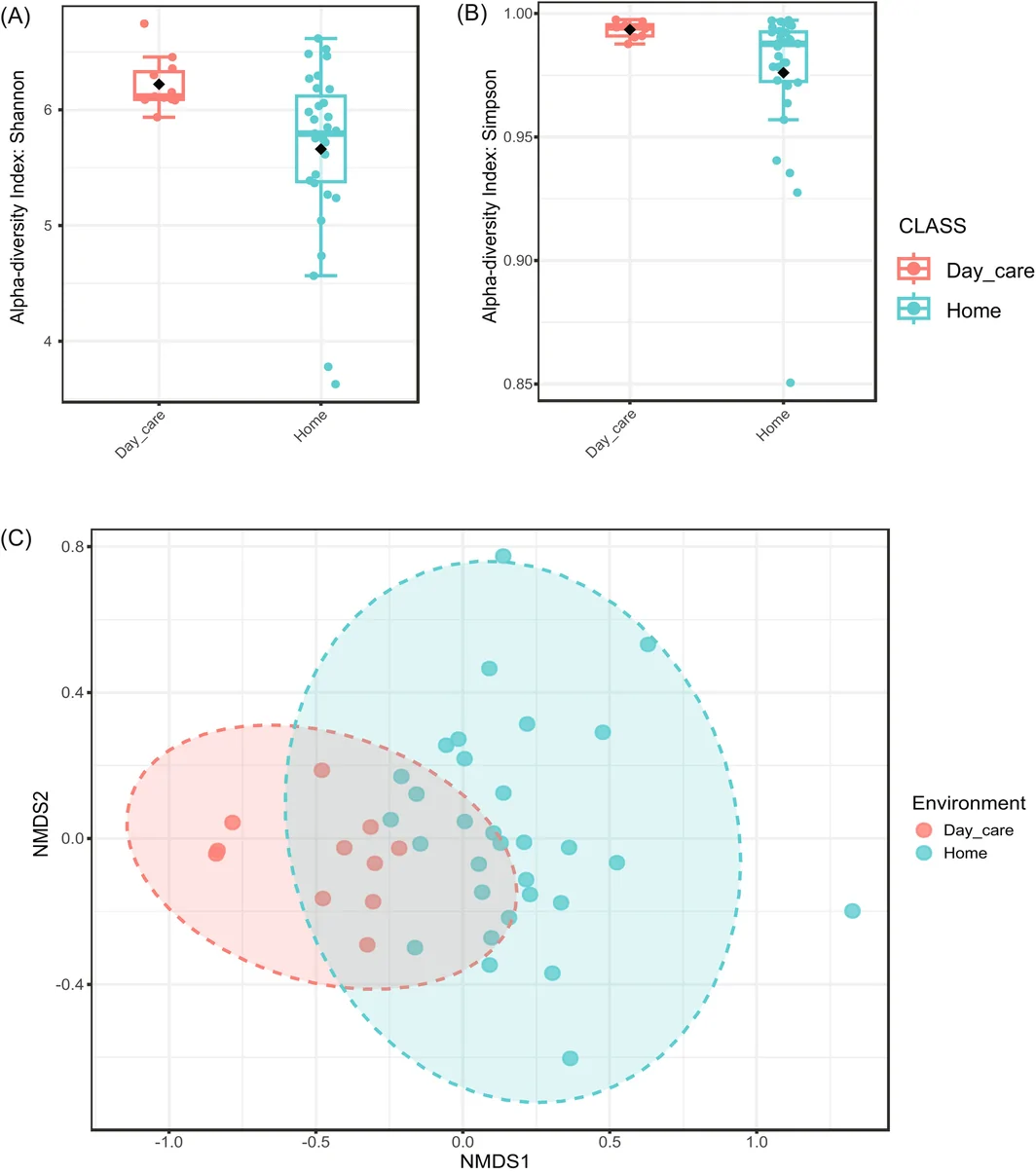
α‐Diversity in daycare and home dust samples using (A) Shannon and (B) Simpson diversity indices; (C) NMDS ordination of β‐diversity (Bray–Curtis dissimilarity). Samples are coloured by location type.

*Pseudomonadota* was the most abundant phylum (12.7%–83.8% abundance) with comparable average prevalence both in daycare and home samples (30.3%–30.7%). Other major phyla were *Actinomycetota* (5.1%–39.1% abundance), *Bacillota* (0.5%–44.7% abundance), *Bacteroidota* (2.0%–17.7% abundance), *Cyanobacteriota* (2.6%–34.3% abundance) and *Deinococcota* (0.8%–13.1% abundance). Altogether, these six phyla accounted for > 96% of the diversity in dust samples from home and daycare environments (Figure 2A), which is in agreement with our previous findings (McKay et al. 2024). On average, members of the phylum *Bacillota* were > 2.5‐fold more prevalent in the home dust than in daycare, whereas members of the phyla *Bacteroidota*, *Cyanobacteriota* and *Deinococcota* were relatively (1.6‐ to 1.9‐fold) more abundant in daycare dust than home samples.

**FIGURE 2:**
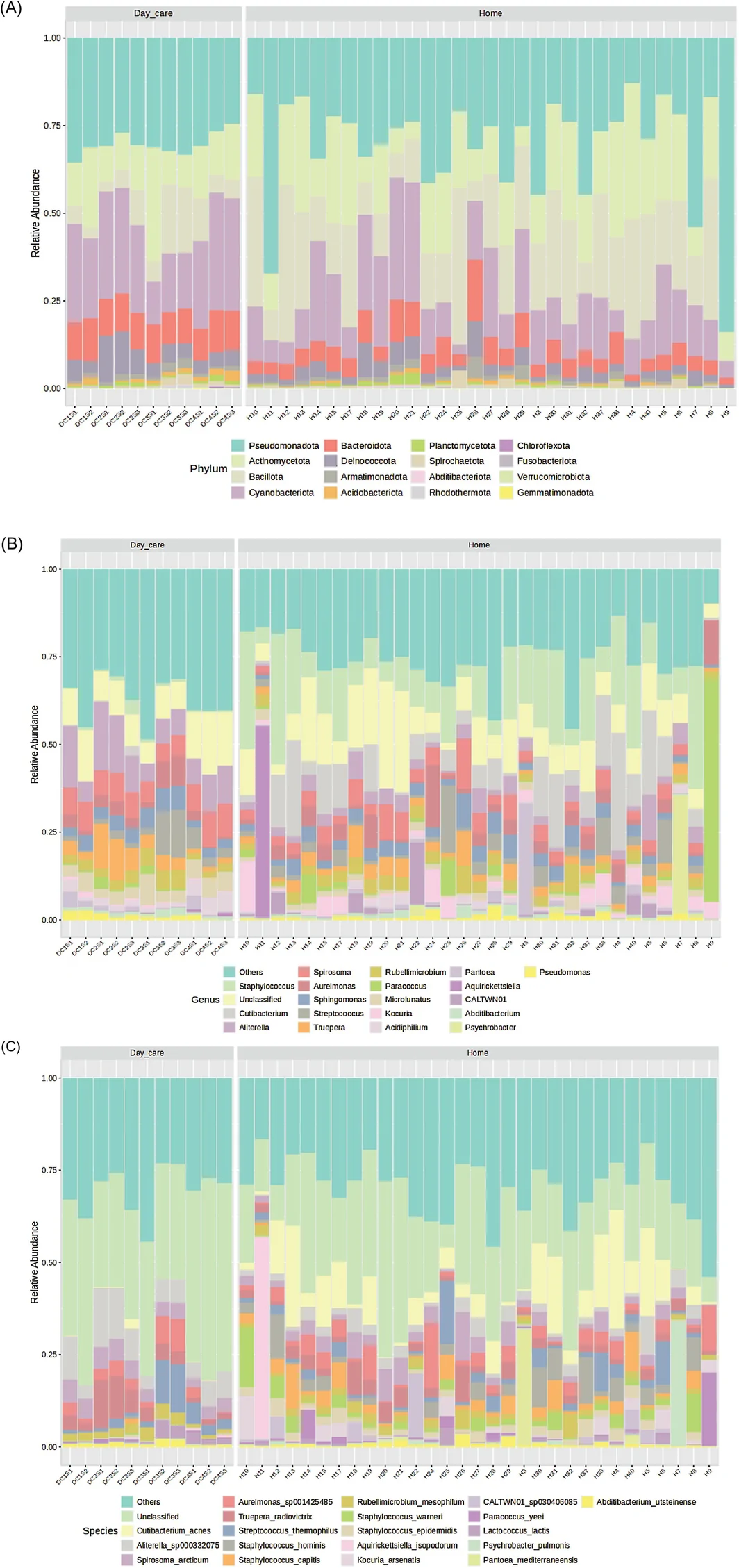
Percentage of taxa abundance in the dust samples from daycares and homes at (A) phylum, (B) genus and (C) species level. A total of 20 most prevalent taxa are displayed, and the remaining taxa are grouped as ‘Others’.

Among the top 18 genera with an average abundance of 1% or higher, members of *Acidiphilium*, *Aliterella*, *Microlunatus*, *Spirosoma* and *Trupera* were 1.8‐ to 3.3‐fold more prevalent in daycare samples than home samples, whereas *Aquirickettsiella*, *Cutibacterium*, *Kocuria*, *Paracoccus*, *Psychrobacter* and *Staphylococcus* were 3.5‐ to 25.4‐fold more abundant in the home samples than daycare (Figure 2B). Sequencing of the full‐length 16S rRNA gene allowed identification of several taxa down to the species level, including many taxa with undefined genus and/or species designations from the GTDB database, such as *Aureimonas* sp001425485 and CALTWN01 sp030406085 (Table S1). Among the top 20 species in the dataset (Figure 2C), the average prevalence of *Aliterella* sp000332075 and *Lactococcus lactis* was more than twofold higher in dust samples from daycares than homes, while *Aquirickettsiella isopodorum*, CALTWN01 sp030406085, *C. acnes*, *Kocuria arsenatis*, *Pantoea mediterraneensis*, *Paracoccus yeei* , *Psychrobacter pulmonis* , *Staphylococcus capitis* , *Staphylococcus epidermidis* , *Staphylococcus hominis* and *Staphylococcus warneri* were up to 77 times more abundant in home dust.

### 2.2 Common and Environmental Specific Microbiome

Of the 3709 filtered ASVs, 3102 were shared, and the distribution of 993 was significantly different between the two environments (univariate analysis, *p* < 0.05). A total of 1472 ASVs were unclassified, accounting for approximately 40% of variants, suggesting a considerable proportion of microbes in indoor environments are yet to be characterised. Dust samples from both environments are quite diverse, with only six genera, *Aliterella*, *Aureimonas*, *Sphingomonas*, *Spirosoma*, *Rubellimicrobium* and *Truepera*, being core, that is, present in at least 80% of samples across both environments (Figure 3A–C). Three additional genera, *Acidiphilium*, *Microlunatus* and *Streptococcus*, were core to the dust samples from daycare (Figure 3B), and *Cutibacterium* and *Staphylococcus* were common in the home dust samples (Figure 3C). At the species level, *Aliterella* sp000332075, *Rubellimicrobium mesophilum* , *Spirosoma arcticum* and *Truepera radiovictrix* were consistently present across most samples in both environments (Figure 3D–F). *Aureimonas* sp001425485 was additionally conserved in dust samples from daycare settings (Figure 3E).

**FIGURE 3:**
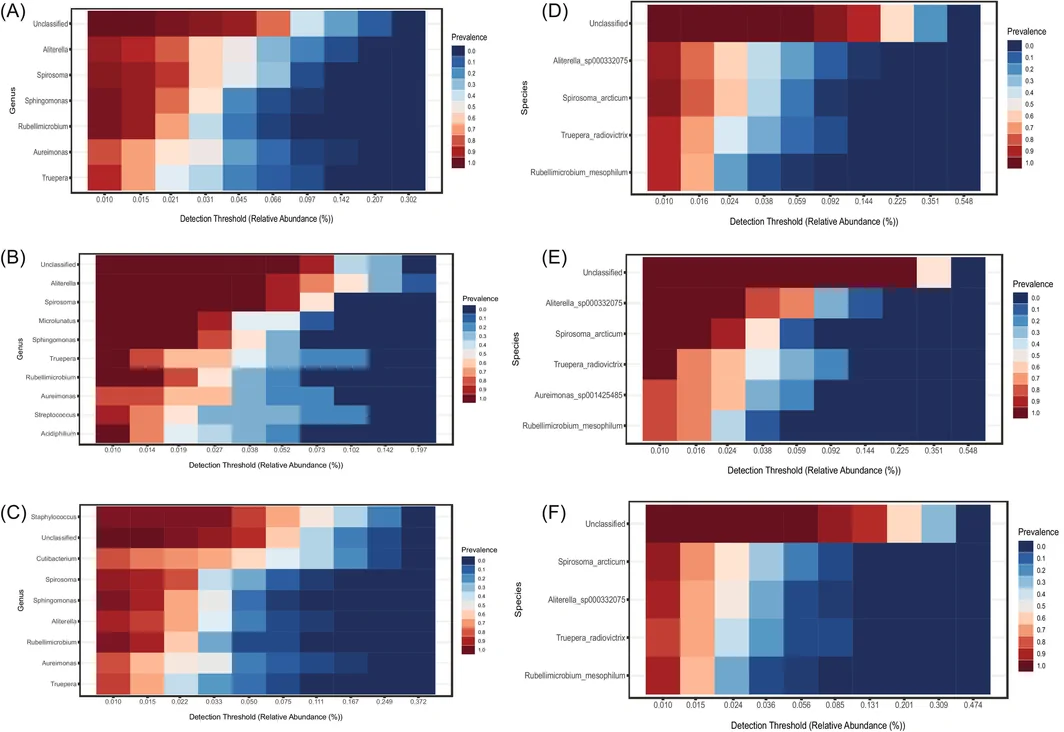
Core taxa with 80% prevalence and at least a 0.01% detection threshold in dust samples at the genus level in (A) both environments, (B) daycare dust, (C) home dust and at the species level (D) both environments, (E) daycare dust and (F) home dust.

A total of 189 species were differentially present between the two environments, including *Arthrobacter oxydans* , *Dankookia rubra*, *Microlunatus aurantiacus* , *Microlunatus panaciterrae* , *Nocardioides glacieisoli*, *Pantoea eucrina*, *Segetibacter koreensis* , *Spirosoma endophyticum* and *Terracoccus luteus* being significantly more prevalent in dust from daycare providers and *C. acnes*, *Corynebacterium tuberculostearicum* , *Staphylococcus. epidermidis* , *S. hominis* and *S. warneri* more abundant in dust from homes (Figure 4 and Table S1). Linear discriminant analysis effect size (LEfSe) analysis confirms differential abundance of genera *Acidiphilium*, *Aliterella*, *Calothrix‐PCC_6303*, *Deinococcus*, *Hymenobacter*, *Lactococcus*, *Microlunatus*, *Nocardioides*, *SCTD01*, *Sphingomonas*, *Spirosoma*, *Streptococcus* and *Truepera* in daycare samples and *Aquirickettsiella*, *CALTWN01*, *Corynebacterium*, *Cutibacterium*, *Kocuria*, *Paracoccus*, *Psychrobacter* and *Staphylococcus* in home samples (Figure 5A). When abundance of bacterial species was compared, *Aliterella* sp000332075, *Streptococcus thermophilus* and *T. radiovictrix* were significantly more abundant in daycare samples, whereas *A. isopodorum*, *CALTWN01 sp030406085*, *C. acnes*, *K. arsenatis*, *P. mediterraneensis*, *P. yeei* , *P. pulmonis* , *S. capitis* , *S. epidermidis* , *S. hominis* and *S. warneri* were prevalent in home dust (Figure 5B). These findings are consistent with environmental taxa being predominant in daycare settings and human‐associated bacteria in household dust samples (Park et al. 2021; McKay et al. 2024).

**FIGURE 4:**
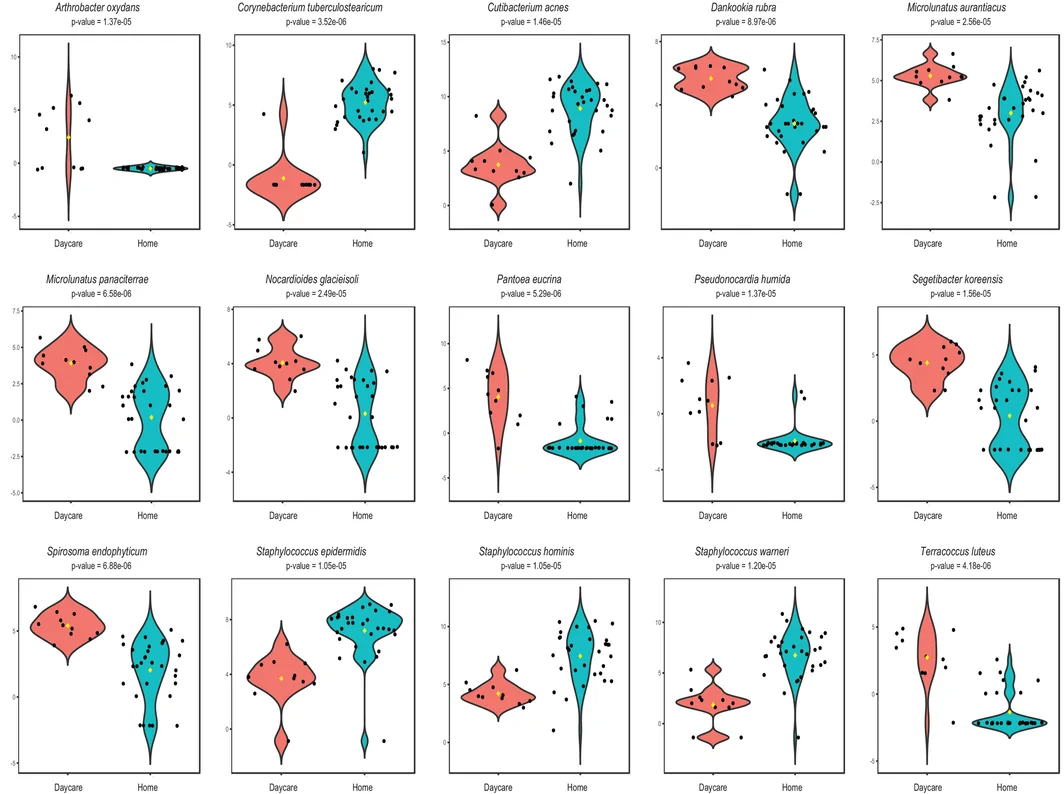
Violin plots showing 12 taxa with significant differences in their abundances between daycare and home dust samples (see Table S1). Log‐transformed filtered counts are shown on the *Y*‐axis.

**FIGURE 5:**
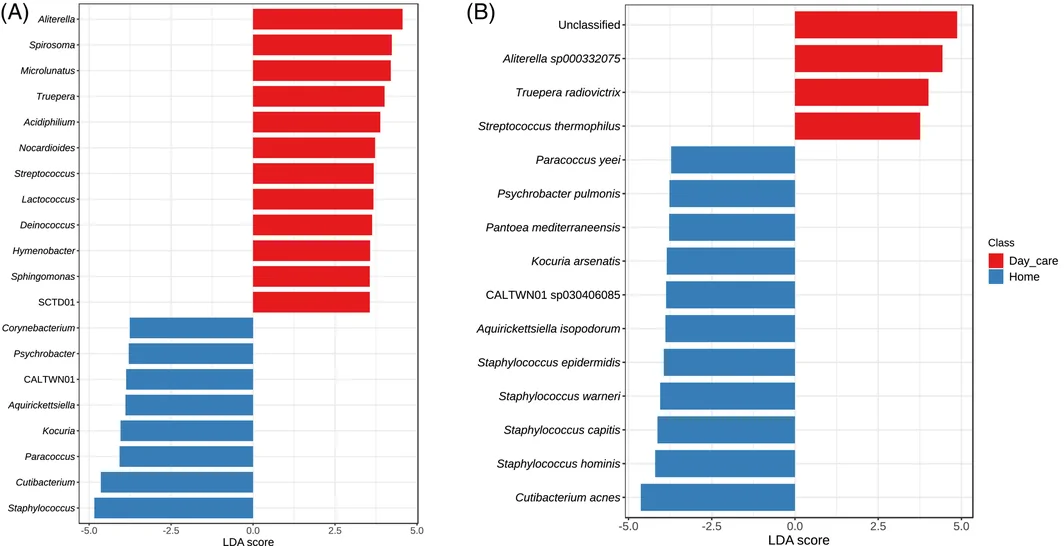
Most prevalent taxa differentiating dust samples from (A) daycares and homes at the genus level and (B) daycares and homes at the species level.

### 2.3 Influence of Pets on Dust Microbiome

We collected 31 dust samples from homes, with 11 of them reporting cats and 5 reporting dogs as pets present at home. Two homes with cats also reported a snail and a fishpond, respectively, and one home with a dog reported guinea pigs, a tortoise and aquarium fish as additional pets. A total of 15 households did not report any pets. Interestingly, microbial diversity between homes with pets and those without pets was comparable (Figure S1A,B). There was no significant difference in α‐diversity (*p* = 0.389 for the Shannon index and *p* = 0.320 for the Simpson index). Microbial composition of homes with or without pets completely overlapped (Figure S1C). However, three species, *Fusobacterium perfoetens* , *Megamonas rupellensis* and *Peptacetobacter hiranonis*, were more abundant in homes with dogs as pets (FDR‐adjusted *p* = 0.002).

### 2.4 Artificial Intelligence (AI)‐Assisted Ecological Profiling of Microbial Diversity

Among the detected species in both environments, 516 were validly published, and we used an AI‐assisted approach to classify them as host‐associated (animal or human), environmental (plant, food, soil, water, etc.) or pathogenic (Table S2 and Figure 6). A total of 12 species were classified as pathogens (5 human, 4 animal and 3 plant‐associated) that constitute only 2.6% of relative abundance in homes and 1.0% in daycares. All 12 pathogens were detected in homes and 10 in daycares (Table 1). Human pathogens included *Clostridium perfringens* , *Moraxella catarrhalis* , *Streptococcus pneumoniae* , *Escherichia coli/shigella* and *Haemophilus influenzae*; plant pathogens were *Agrobacterium rubi* , *Clavibacter insidiosus* and *Xanthomonas oryzae*; and animal pathogens included *A. isopodorum*, *Brachyspira hampsonii*, *Corynebacterium auriscanis* and *Staphylococcus pseudintermedius* (Table S2). Only *C. auriscanis* and *X. oryzae* were not detected in daycare samples. Notably, the two respiratory human pathogens *S. pneumoniae* and *H. influenzae* were more abundant in daycares than homes (≈2.4‐ and 3.5‐fold), consistent with a previous study and a possibility of child‐to‐child respiratory transmission (Sa‐Leao et al. 2008). The enteric ( *E. coli* /*Shigella* and *C. perfringens* ) and plant/animal pathogens leaned towards homes, potentially contributed by local hygiene practices, raw food (plant‐based and animal meat) handling and domestic pets in the indoor environment (Flores et al. 2013; Fuhrmeister et al. 2019; Daly et al. 2024; Paulos et al. 2025).

**FIGURE 6:**
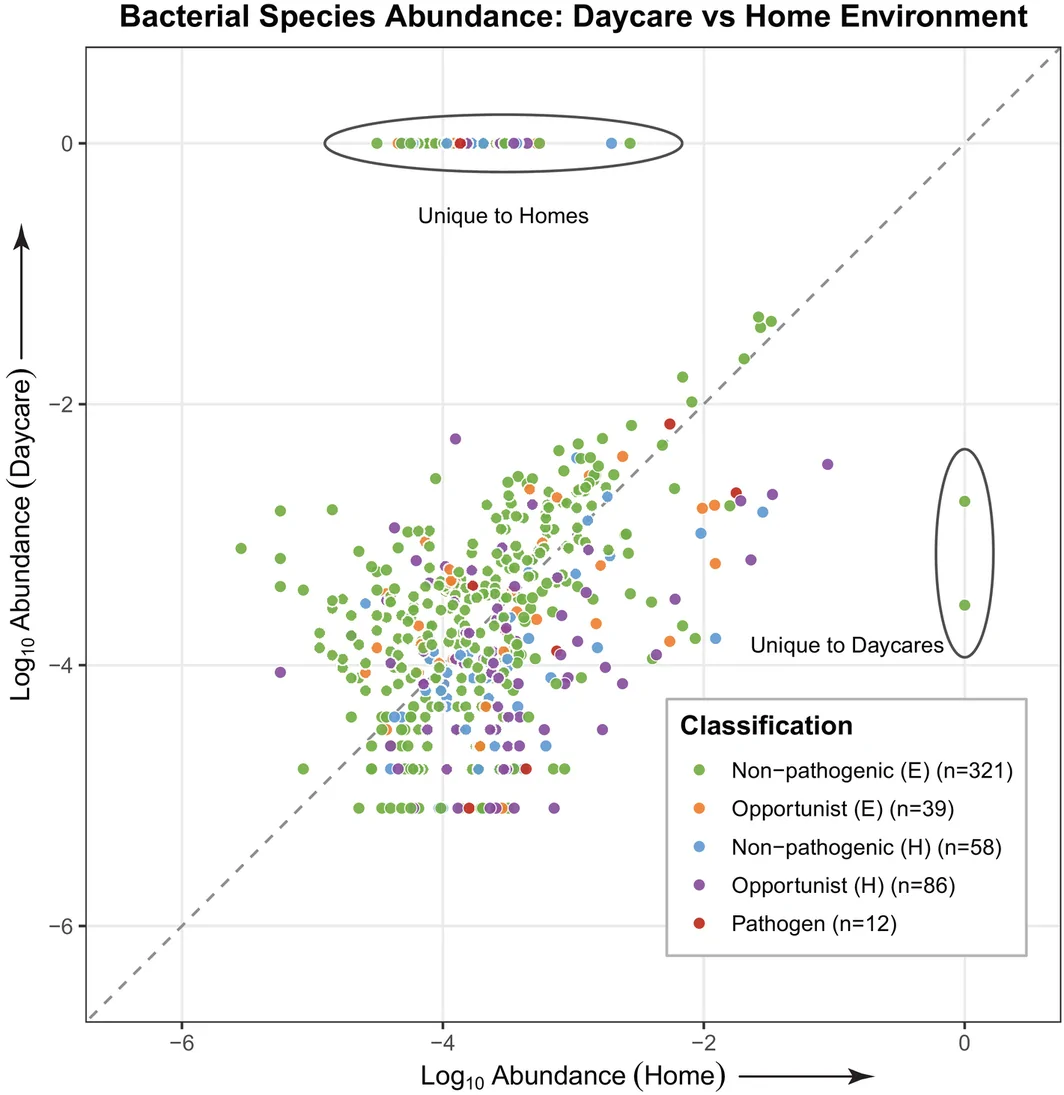
Relative abundance at a log_10_ scale of non‐pathogenic, opportunistic pathogens and pathogens in daycare and home environments. Arrows show increasing abundance from −5 to −1 on both axes, and taxa unique to each environment are labelled. E, environmental taxa; H, host‐associated taxa.

**TABLE 1:** Overall abundance (%) of AI‐assisted classified species between daycare and home dust samples.

Characteristic	Daycare	Home
Host‐associated	1.5 (46)	7.0 (58)
Host‐associated (opportunistic)	2.7 (73)	20.4 (86)
Environmental	35.6 (288)	27.7 (319)
Environmental (opportunistic)	2.2 (33)	5.3 (39)
Pathogen	1.0 (10)	2.5 (12)
Uncharacterised species	18.7 (80)	13.8 (91)
Unclassified taxa	38.2	23.4

*Note:* The number of species is mentioned in parentheses, where available.

A total of 144 species were identified as host‐associated (excluding pathogens), with 86 of these having potential to cause opportunistic infections (Table S2). All 144 host‐associated species were detected in home dust (overall abundance 27.4%), but only 119 of these were present in the daycare environment in much lower abundance (overall abundance 4.2%; Table 1). A total of 360 species were identified as environmental, including 39 with the ability to cause opportunistic infections. 321 species of these were detected in daycare dust and 358 in home dust samples (Table 1). Although more environmental species were detected in dust from homes than daycares, the abundance of environmental taxa was relatively higher in daycare (37.8%) than home dust (32.9%; Table 1). Daycare samples also had a higher proportion of uncharacterised and unclassified species than home samples (Table 1). Interestingly, all 137 species identified as pathogenic or opportunistic pathogens were detected in home environments, but only 85% of them were present in daycare dust.

Two environmental species, *Pseudarthrobacter oxydans* and *Mixta theicola*, were only detected in daycare samples in very low abundance. *P. oxydans* is known for its abilities to promote plant growth (Oubohssaine et al. 2022) and absorption of heavy metals (Mechirackal Balan et al. 2018). A total of 66 species, including 25 host‐associated (13 opportunistic pathogens), 39 environmental (6 opportunistic pathogens) and 2 pathogens, were unique to home dust samples. Among the species unique to home samples are the dairy‐associated bacteria *Brachybacterium tyrofermentans* , *Brevibacterium sandarakinum* , *Brevibacterium aurantiacum* , *Corynebacterium kefirresidentii* and *Microbacterium lacticum* , further corroborating that raw food handling in indoor environments is an important source of introducing microbial diversity (Table S2). *Sphingomonas molluscorum* and *Kocuria marina* are present in the marine environment, and the presence of these species in the indoor environment may be associated with seafood (Uzair et al. 2018; Romanenko et al. 2007). Among the home‐specific species are *Ezakiella coagulans*, *which* is often a part of the healthy human vaginal microbiome (France et al. 2022, 2020) and *Prevotella bivia*, *which* is an important opportunistic pathogen of the female urogenital tract (Abat et al. 2016; George et al. 2024). Two commensal bacteria associated with vaginal health, including *Gardnerella vaginalis* and *Lactobacillus crispatus* , were detected in both environments; the former can be an important opportunistic pathogen (Harwich Jr. et al. 2010), whereas the latter is an important vaginal probiotic and is commercially available on the market (Mandar et al. 2023).

## 3 Discussion

### 3.1 Exposure to a Diverse Microbiome in Daycare and Home Settings

Home and daycare environments are rich in microbial diversity (Figure 1A–C), particularly species belonging to the genera *Actinomycetota*, *Bacillota*, *Bacteroidota*, *Cyanobacteriota*, *Deinococcota* and *Pseudomonadota* (Figure 2A–C). These findings are consistent with previous studies analysing microbial diversity in dust samples collected from home, daycare and school environments (Park et al. 2021; Nygaard and Charnock 2018; McKay et al. 2024). *Halospirulina* was reported to be the most abundant genus among the dust samples from elementary schools in Philadelphia, USA (Park et al. 2021), which was not detected in our study.

Interestingly, microbial diversity and composition of microbial communities in daycare and household dust are comparable between samples collected during the COVID‐19 pandemic (McKay et al. 2024) and post‐pandemic (Figures 1 and 2). Microbial communities were more heterogeneous, with a higher number of bacterial taxa detected in home dust when compared to dust samples from daycares (Figure 1C). Our results also show that environmental taxa were more prevalent in daycare settings, whereas human‐associated bacteria were more prevalent in dust from homes (Figure 5A). Although these findings are consistent with our previous results (McKay et al. 2024), they contradict other reports of human‐associated taxa being most abundant in childcare settings (Nygaard and Charnock 2018; Beasley et al. 2022). This potentially indicates that there is still more emphasis on personal hygiene and robust cleaning practices in UK daycare settings post‐COVID‐19 pandemic. In addition, outdoor activities in the daycare settings may be contributing to a higher prevalence of environmental taxa.

An analysis of full‐length 16S rRNA gene sequences offered more resolution in terms of identification of taxa down to the species level that was not possible from analysing partial 16S rRNA gene in our previous study (McKay et al. 2024). For example, we previously reported only two taxa, the genus *Nocardioides* and an unclassified member of the family *Gemmatomonadaceae*, differentially abundant in daycare environments and only *Staphylococcus* associated with home dust (McKay et al. 2024). In this study, we report additional environmental genera, including *Acidiphilium*, *Aliterella*, *Deinococcus*, *Hymenobacter*, *Microlunatus*, *Sphingomonas*, *Spirosoma* and Truepera, food‐associated *Lactococcus* and human‐associated *Streptococcus* in daycare and additional human‐associated taxa *Corynebacterium*, *Cutibacterium* and *Kocuria*, along with *Aquirickettsiella*, *Paracoccus* and *Psychrobacter* in home settings (Figure 5A). At the species level, 3 environmental species were associated with daycare and 11 species, mostly human‐associated, with home environments (Figure 5B). For example, *C. acnes* is differentially associated with the home environment (Figures 4 and 5B), which correlates with adolescents and young adults being home occupants (Nygaard and Charnock 2018). Conversely, *C. acnes* was markedly less abundant in samples from daycares attended predominantly by pre‐pubescent children (Leyden et al. 1975).

In addition to human activities and occupancy levels, the presence of pets at homes can also influence microbial diversity (Tan et al. 2025; Guo et al. 2026; Chawla et al. 2023). In our study, microbial diversity was not affected by the presence of cats or dogs as pets (Figure S2A,B); however, *F. perfoetens* , *M. rupellensis* and *P. hiranonis* were more abundant in homes with dogs. *F. perfoetens* and *P. hiranonis* are part of the canine gut microbiome (You and Kim 2021; Correa Lopes et al. 2024; Doshier et al. 2026), with the latter offering protection from diarrhoea and being commercially available as a pet probiotic. While the impact of the interaction of these species on human health is yet to be characterised, *M. rupellensis* was found to be associated with obesity due to enhancing intestinal lipid absorption (Wu et al. 2024).

Notably, a substantial proportion of microbial diversity is unclassified (Table 1 and Table S1). Indeed, only < 10% of estimated prokaryotic diversity is known either via culture‐based methods (< 22,000 species) or metagenomic characterisation of environmental samples (~135,000 species) (Jiménez et al. 2026; Nouioui and Sangal 2022; Wu et al. 2025). This finding highlights a critical knowledge gap in the current understanding of microbial ecology in indoor environments, their interaction with humans and potential impact on human health.

### 3.2 How Prevalent Are Pathogens in Indoor Environments?

Various pathogens have been reported in indoor environments, emphasising the risk of exposure and chronic infections to occupants (Guo et al. 2026; Chawla et al. 2023; Jarma et al. 2024; Liu et al. 2022; Leung et al. 2019). Only 2.3% (12/516) of the known bacterial species were classified as pathogenic (excluding uncharacterised species) in this study (Figure 6 and Table S2). 27.9% of the bacterial species were identified as host‐associated and 69.9% were identified as environmental. 24.3% (125/516) of host‐associated and environmental species were predicted to be opportunistic pathogens (Figure 6; Table 1 and Table S2).

Pathogenic and opportunistic pathogens were more prevalent in the home settings (all 137 species) when compared to the daycare environment (116/137 species; 84.7%). These findings contradict the report of equal abundance of pathogenic taxa in the home environment (Jarma et al. 2024). *Acinetobacter*, *Anaerococcus*, *Corynebacterium*, *Legionella*, *Mycobacterium*, *Pseudomonas*, *Sphingomonas*, *Staphylococcus* and *Streptococcus* were defined as pathogen‐associated taxa (Jarma et al. 2024), but not all species in these genera are pathogenic. The five most abundant species and ~50% of differentially abundant species in indoor air were identified to be pathogenic by full‐length 16S rRNA gene sequencing (Liu et al. 2022). The detected species in indoor air were much lower in number than the taxa identified in the indoor dust in this study. The variation in the number and nature of the microbiome is likely contributed to by the difference in the sample types (Fu et al. 2025). Settled dust provides a more comprehensive insight into microbial diversity in the indoor environment that may be contributed not just by the occupants but also by their activities, such as outdoor walks. Other factors, including ventilation, may also introduce microbes from outdoor air to indoor environments.

Among the detected pathogens, *C. perfringens* is one of the important pathogens that can infect both humans and livestock, causing a variety of infections from food‐borne enteritis and enterocolitis to wound infections (gas gangrene) (Mehdizadeh Gohari et al. 2021). We also detected *S. pneumoniae* , *H. influenzae* and *M. catarrhalis* , which are the most common pathogens of the upper and lower respiratory tract with the potential to cause chronic obstructive pulmonary disease (Schaar et al. 2011). Various *E. coli* and *Shigella* strains can cause severe gastroenteritis, especially among young children (Buskirk et al. 2022). A number of species identified as opportunistic pathogens are also important human pathogens, including *Acinetobacter baumannii* , *Corynebacterium kroppenstedtii* , *Corynebacterium minutissimum* , *Corynebacterium urealyticum* , *Streptococcus anginosus* , *Staphylococcus aureus* and *Staphylococcus lugdunensis* (Table S2). *C. kroppenstedtii* has been associated with granulomatous lobular mastitis in women but can also cause various other infections (Brouwer de Koning et al. 2022; Sasi et al. 2025; Tauch et al. 2016). *C. urealyticum* can infect the urinary tract and can also cause other invasive infections that could be life‐threatening (Salem et al. 2015). *S. pseudintermedius* can cause a range of zoonotic infections, including skin, ear and wound infections in humans (Roberts et al. 2024).

Among the pathogenic taxa, three species, *A. rubi* , *C. insidiosus* and *X. oryzae* , are plant pathogens (Sicard et al. 2025; EPPO 2022; Vargas Ribera et al. 2024). *B. hampsonii* is an animal pathogen, primarily infecting pigs (Mirajkar et al. 2016) and *A. isopodorum* can infect crustaceans (Wang and Chandler 2016). Although *A. isopodorum* and *S. pseudintermedius* are animal pathogens, these bacteria are capable of causing life‐threatening zoonotic infections in humans (Bygott et al. 2008; Subedi et al. 2021).

Overall, 22.6% (137 out of 607) of identified and uncharacterised species diversity were classified as pathogenic or opportunistic pathogens, accounting for 6.9% and 28.2% abundance in daycare and home environments, respectively (Table 1). This indicates that both indoor environments are dominated by non‐pathogenic, largely environmental taxa; however, home environments carried a markedly higher relative load of (predominantly human‐associated) pathogens. Low microbial heterogeneity and fourfold lower abundance of pathogenic bacteria in daycare settings potentially indicate adherence to robust cleaning/hygiene practices in the post‐COVID era. In addition, some of these differences may be due to the frequency of cleaning and the duration of dust being accumulated in the vacuum bags, which may be longer in homes than in the daycare settings. Domestic dust integrates continuously shed skin squames, sebum and oral/respiratory and gut‐derived microbiota from resident occupants (including pets) and those contributed by other activities such as dust brought back from outdoor walks (Tan et al. 2025; Guo et al. 2026; Chawla et al. 2023). Therefore, household vacuum dust can integrate deposition over weeks to months, whereas daycare samples are likely to represent a comparatively shorter accumulation window owing to more frequent institutional cleaning. Standardising the dust‐accumulation interval, recording time‐since‐last‐cleaning and applying viability‐resolved approaches would help disentangle genuine ecological differences from accumulation‐driven artefacts in future work.

### 3.3 Immunomodulatory and Immunoregulatory Microbiome

We previously reported several bacterial taxa both in daycare and home environments that may help decrease the risk of non‐communicable and infectious diseases among children (McKay et al. 2024). Full‐length 16S rRNA amplicon sequencing allowed us to identify many species that can offer such protection in both environments. Multiple *Acinetobacter* species were detected in dust samples from both environments (Table S1), with some, such as *Acinetobacter lwoffii* , being able to confer protection against environmental allergens and inflammation (Fyhrquist et al. 2014). *Akkermansia muciniphila* is reported to induce immunity against multiple diseases, including diabetes, liver steatosis and obesity (Cani et al. 2022; Depommier et al. 2019). Many species in the gut microbiome, such as *Bifidobacterium animalis* and *Lactobacillus acidophilus* , influence T lymphocyte function, which involves secretion of various cytokines (interferon‐γ, interleukins including IL‐17 and IL‐22) by pro‐inflammatory T helper cells and modulating anti‐inflammatory responses via Foxp3+ regulatory T cells (Cristofori et al. 2021). *Bifidobacterium longum* , *Bifidobacterium bifidum* and *B. animalis* play a critical role in developing immunity against various diseases, including cancers and tumours, in neonates (Chen, Wu, et al. 2025; Ennis et al. 2024; Turroni et al. 2019; Lee et al. 2021; Chen, Li, et al. 2025).

We detected 16 *Corynebacterium* species in dust samples, and some of them can induce immunity against pathogenic bacteria. For example, *Corynebacterium pseudodiphtheriticum* can modulate the respiratory innate immune response to help protect the upper respiratory tract from various viral and bacterial infections (Kanmani et al. 2017). *C. tuberculostearicum* plays an important role in human skin health via the canonical nuclear factor‐κB inflammatory signalling pathway (Altonsy et al. 2020). A reduction in the prevalence of *Faecalibacterium*, including *Faecalibacterium prausnitzii*, is associated with several conditions, including inflammatory bowel disease, colorectal cancer, dermatitis, depression and other gastrointestinal diseases or disorders (Martin et al. 2023). *Faecalibacterium* species are emerging as the next‐generation probiotic. *Lacticaseibacillus rhamnosus* has been involved in inducing secretion of various cytokines and protection against atopic dermatitis/eczema and asthma, and improving outcomes for mechanically ventilated patients (Kalliomaki et al. 2007; Szyller et al. 2025). It has become a commonly used probiotic abundantly available on the market (Yang et al. 2025). Similarly, *L. lactis* is involved in anti‐allergy and anti‐inflammatory activities that can significantly modulate conditions such as atopic dermatitis (Baek et al. 2023). As mentioned previously, *L. crispatus* plays an important role in vaginal health and is reported to reduce the volume and odour of vaginal discharge and vulvovaginal irritation in patients with bacterial vaginosis or vulvovaginal candidiasis (Mandar et al. 2023). Commensal *S. epidermidis* produces protective ceramides that are important for skin barrier homeostasis and enhance protection against intracellular *S. aureus* via activation of gamma‐delta T cells and perforin‐2 induction (Pastar et al. 2020; Zheng et al. 2022). *S. lugdunensis* also suppresses *S. aureus* growth and is a potential probiotic species (Zhang et al. 2025). *Streptococcus mitis* induces cross‐reactive antibodies that can protect against *S. pneumoniae* (Engen et al. 2014; Shekhar et al. 2019). Therefore, several bacterial species detected in dust from daycare and home environments likely contribute to inducing immunity against various communicable diseases and NCDs.

### 3.4 Reliability and Limitations of Bacterial Classification

Our approach to classify detected species is evidence‐based and reproducible. We identified non‐pathogenic, opportunistic pathogens and frank pathogens by extracting information on their virulence potential and ecological origin (environmental or host‐associated) using curated authoritative taxonomy and pathogen resources, including BacDive, List of Prokaryotic names with Standing in Nomenclature (LPSN), the WHO Bacterial Priority Pathogens List and the EPPO and WOAH registers (see Section 5 and File S1). This independent consideration of pathogenic potential and ecological origin avoids erroneous conclusions based solely on clinical severity or habitats; that is, isolation of a microbe from a clinical specimen such as blood, wound or catheter and so forth may not reflect its dominant isolation source, which may be environmental. A bare infection report or a single incidental clinical isolate of an otherwise environmental or probiotic species is sufficient evidence of opportunism. These conservative safeguards reduce over‐calling of species into different categories. Any borderline or mixed‐profile taxa were curated with the rationale based on the published literature. All species discussed in this article were correctly identified by an AI‐assisted approach and were manually verified against the existing literature. This approach also corrected the nomenclature of multiple species to validly published names (Table S2). Therefore, the AI‐assisted classification approach for bacterial species used in this study is highly robust and reliable.

However, this approach relies on the accuracy of information in these databases, and many species, such as GTDB‐only lineages, are often not frequently isolated or characterised. There is a possibility that the pathogenic potential of a rare or uncommon species is incorrectly recorded based on a single isolate from a non‐clinical source and vice versa. Some species may be non‐pathogenic but may include some highly virulent strains; for example, *S. aureus* is primarily a commensal, but many strains, such as USA300 MRSA, are highly virulent (Tigabu and Getaneh 2021; Di Bella et al. 2025). Based on our approach, it is recorded as an opportunistic pathogen, but we cannot confirm whether the data are derived from commensal or highly virulent strains based on 16S rRNA sequence. Furthermore, the recovered sequence will not allow us to distinguish living cells from non‐viable material such as dead and lysed bacteria; extracellular ‘relic’ DNA and DNA carried within microbial extracellular vesicles may all contribute to the detected sequences. For example, *C. acnes* is detected with an average prevalence of 8.9% in the home dust community. As a human skin commensal, shed cells may die due to desiccation in the dust over time and may lyse. The released DNA from dead cells may be captured on the dust particles or may be vesicle‐borne (Jingushi et al. 2024). Therefore, high relative abundance may reflect cumulative shed‐and‐dead material, not a live bacterial population growing in the carpet. Overall, these classification assignments describe DNA‐based presence and relative abundance and do not establish viability, infectivity, dose or actual disease risk. These ‘potential pathogens’ should not necessarily mean evidence of hazard. Therefore, this classification is best regarded as a reproducible, database‐anchored snapshot of pathogenic and ecological potential to guide interpretation.

## 4 Conclusions

Dust samples from daycare and home environments are rich in diverse microbes, but home environments are more heterogeneous, encompassing a higher number of bacterial taxa in comparison to daycare. Human‐associated taxa, including pathogenic bacteria, are more abundant in homes, whereas daycare settings have a higher prevalence of environmental taxa, which may reflect continued rigorous hygiene practices post‐COVID‐19 pandemic in daycares and a combination of indoor and outdoor activities. Cats and dogs as pets do not significantly influence household microbial diversity; however, three species, including two belonging to the canine gut microbiome, were more abundant in homes with dogs as pets. Finally, many bacterial species with abilities to induce immunity against a variety of infectious diseases and NCDs, including asthma, allergies and cancers and tumours in neonates, are present in both environments and therefore, exposure to these may provide protection against various diseases.

## 5 Materials and Methods

### 5.1 Sample Collection

Vacuum bags or dust subsamples were collected along with the information on the occupants, including the number, gender and ages of occupants from 31 homes between April and July 2024, as previously described (McKay et al. 2024; Thompson et al. 2021). Three sequential dust samples were collected a month apart from three local daycare providers, and two samples were collected from an additional daycare (2 months apart) between May and July 2024. In total, 42 samples were collected, 31 from homes and 11 from daycare providers.

### 5.2 DNA Extraction and Amplicon Sequencing

DNA was extracted from dust samples as previously described (McKay et al. 2024). Dust samples were received in various types of bags, including plastic bags, multi‐layer paper or synthetic microfibre vacuum bags. One end of each bag was cleaned with a Virkon‐sprayed wipe and was cut open using a sterile, single‐use scalpel in a biosafety cabinet. The dust from the interior core of the bag, making sure that there was no drag against the outer, untreated exterior surface, was sieved through a UV‐sterilised 250 μm single‐use nylon mesh to remove larger particulates and fibrous material. DNA was extracted from 1 g dust using the DNeasy PowerSoil Pro Kit (Qiagen), and an extraction control was included in each batch. Full‐length 16S rRNA amplicon sequencing was carried out using the PacBio Revio instrument by the NU Omics facility at Northumbria University.

### 5.3 Data Analyses

16S amplicon sequencing reads were analysed using HiFi‐16S‐workflow. Briefly, Cutadapt (Martin 2011) and SeqKit (Shen et al. 2016) were used to pre‐process the data. Trimmed reads were analysed using QIIME2 (Bolyen et al. 2019) and DADA2 (Callahan et al. 2016) with a maximum expected error threshold for read filtering (max_ee) of 7 and the default omegac value of 1e−40 to identify ASVs. ASVs were classified using VSEARCH (Rognes et al. 2016) and a Naive Bayes classifier with Greengenes2 (McDonald et al. 2024), GTDB (Parks et al. 2020, 2018) and Silva (Yilmaz et al. 2014; Quast et al. 2013) databases. The pipeline uses MAFFT (Katoh and Standley 2013) for sequence alignment and FastTree (Price et al. 2010) for constructing phylogenetic trees. The Naive Bayes classifier approach classified a higher percentage of ASVs at the genus and species level. Contaminants introduced during the DNA extraction (extraction controls) and those introduced during the library preparation (mock‐community and negative controls) were removed using the decontam package in R (Davis et al. 2018), which removed 43 ASVs from the biological samples, corresponding to 1.32% of reads.

GTDB‐tk assigns alphabetic suffixes to taxon names to indicate that the original group is not a single evolutionary lineage, for example, *Acinetobacter*_*lwoffii*, *Acinetobacter*_*lwoffii*_O and *Acinetobacter*_*lwoffii*_N. In such cases, polyphyletic suffixes were collapsed to classical binomials for statistical analysis to avoid artificial inflation of species richness. Statistical analysis and visualisation of data were carried out using MicrobiomeAnalyst 2.0 (Lu et al. 2023). In total, 19,566 ASVs were identified, and 12,518 ASVs that were only observed in a single sample and 3339 low‐abundance ASVs (< 10% prevalence and a minimum count of 4) were filtered. The data were rarefied to a minimum sample depth of 11,358 reads that captured most of the diversity in the daycare and home samples (Figure S2A,B). Total sum scaling was used for data normalisation. Shannon and Simpson α‐diversities were calculated using the Mann–Whitney/Kruskal–Wallis method and (post hoc) pairwise comparisons. β‐diversity analysis was performed using Bray–Curtis dissimilarity and the Permutational MANOVA (PERMANOVA) approach, and diversities were visualised with two‐dimensional NMDS ordination. Core taxa with a sample prevalence of 80% and a detection threshold of 0.01 were identified at the genus and species level. Statistically significant differences in taxa abundance between homes and daycare were determined by the Mann–Whitney/Kruskal–Wallis method with FDR adjustment (adjusted *p* value cut‐off 0.001). LEfSe analysis was performed to detect differential abundance at the genus and species level.

Ecological profiling of bacterial species was performed based on their pathogenicity and ecological niche, using a reproducible, rule‐based pipeline (File S1) that drew on authoritative taxonomic and clinical databases, implemented with the assistance of AI (Claude and Anthropic). Each species name was first resolved against the NCBI Taxonomy database (https://www.ncbi.nlm.nih.gov/taxonomy) and the LPSN (Freese et al. 2025), with names absent from these sources checked against the SeqCode database (Hedlund et al. 2022). Information on isolation source, sample type, biosafety level and strain records were retrieved from BacDive (https://bacdive.dsmz.de/) and pathogenic status was validated against public resources including the EMBL‐EBI Pathogens Portal (https://www.pathogensportal.org), WHO Bacterial Priority Pathogens List (World Health Organization 2024) and the EPPO (https://gd.eppo.int) and WOAH (https://www.woah.org/en/what‐we‐do/animal‐health‐and‐welfare/animal‐diseases/) to achieve high accuracy.

Microbial species were classified as ‘pathogens’ if they were primary disease‐causing organisms with dedicated virulence mechanisms causing significant morbidity in immunocompetent hosts; ‘opportunists’ when they cause infection primarily in immunocompromised individuals or specific clinical contexts (medical devices, surgical trauma and microbiota displacement), evidenced by isolation from a genuine clinical or infection specimen, a biosafety level ≥ 2 or clinical priority‐list membership. Species with no documented disease association were classed as ‘non‐pathogenic’. To avoid over‐calling benign organisms, a generic infection‐associated database tag was not treated as evidence of pathogenicity unless supported by a genuine clinical specimen and a single incidental clinical isolate of an organism whose predominant niche was non‐clinical or which was a documented probiotic‐associated commensal; such organisms were not classified as opportunists. Independently, each species was assigned a primary and secondary ecological niche as human, animal, food, environmental or plant, based on the isolation frequency. Species that could not be resolved in any database or for which no isolation evidence was available were curated using searches of PubMed to identify the source of isolation, ecological role and clinical significance. The pathogenicity class, ecological niche, primary and secondary isolation sources, supporting evidence and the specific database records underlying each call are provided in Table S2.

## Author Contributions

**Jill A. McKay:** conceptualization, funding acquisition, methodology, supervision, writing – review and editing. **Richard Harding‐Crooks:** methodology. **Vartul Sangal:** conceptualization, funding acquisition, methodology, software, data curation, formal analysis, supervision, writing – original draft.

## Funding

This study was supported by Northumbria University.

## Conflicts of Interest

The authors declare no conflicts of interest.

## Supporting information


**Figure S1:** Alpha diversity in home dust samples with cats and dogs as pets or no pets using (A) Shannon and (B) Simpson diversity indices; (C) NMDS ordination of β‐diversity (Bray–Curtis dissimilarity).

**Figure S2:** A rarefaction curve showing species richness in rarefied reads (A) in the daycare and (B) in the home samples.

**File S1:** R Script to assign taxa into categories as non‐pathogenic, opportunistic pathogen or pathogen.

**Table S1:** Variation in the abundance of bacterial species in daycare and home environments.

**Table S2:** AI‐assisted classification of known bacterial species.

## Data Availability

The sequencing data generated in this study are openly available from the National Center for Biotechnology Information (NCBI) archive via BioProject accession PRJNA1440349 (https://www.ncbi.nlm.nih.gov/bioproject/1440349).
