# Supplementary material for: What is in the Dust? Species‐Level Profiling Reveals Pathogenic and Protective Potential of Microbial Communities in Daycare and Home Environments
Source: Environ Microbiol Rep. 2026 Oct 14;18(5):e70433. doi: 10.1111/1758-2229.70433 (PMC13668400; doi:10.1111/1758-2229.70433)
Supplement: Supplementary file 1 [file EMI4-18-e70433-s003.pdf]

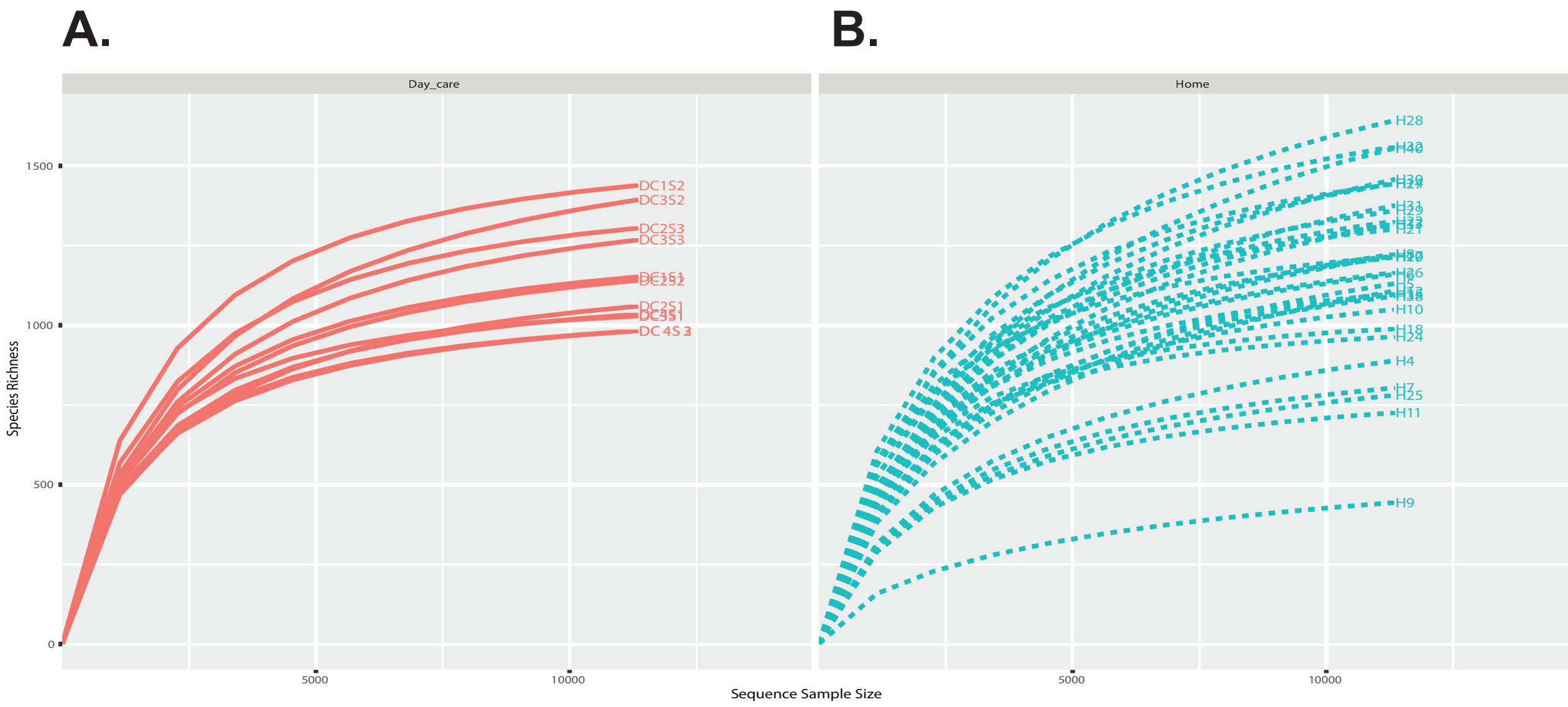

Figure S2. A rarefaction curve of showing species richness in rarefied reads A. in the Daycare and B. in the Home samples.
