# Supplementary material for: What is in the Dust? Species‐Level Profiling Reveals Pathogenic and Protective Potential of Microbial Communities in Daycare and Home Environments
Source: Environ Microbiol Rep. 2026 Oct 14;18(5):e70433. doi: 10.1111/1758-2229.70433 (PMC13668400; doi:10.1111/1758-2229.70433)
Supplement: Supplementary file 2 [file EMI4-18-e70433-s004.pdf]

Lorem ipsum

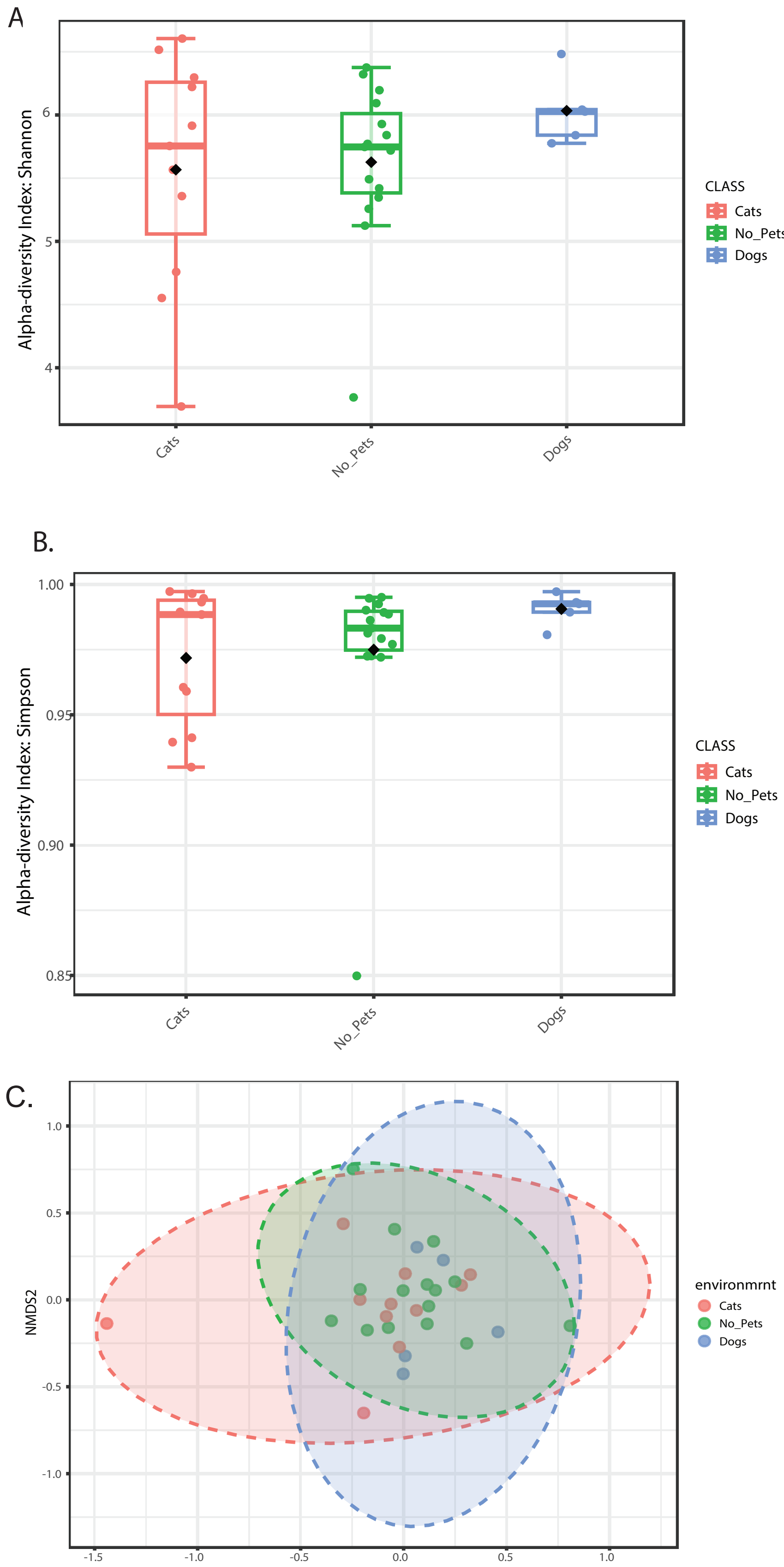

Figure S1. Alpha diversity home dust samples with cats and dogs as pets or no pets using (A) Shannon and (B) Simpson diversity indices; (C) NMDS ordination of β-diversity (Bray–Curtis dissimilarity).
