# Supplementary material for: What is in the Dust? Species‐Level Profiling Reveals Pathogenic and Protective Potential of Microbial Communities in Daycare and Home Environments
Source: Environ Microbiol Rep. 2026 Oct 14;18(5):e70433. doi: 10.1111/1758-2229.70433 (PMC13668400; doi:10.1111/1758-2229.70433)
Supplement: Supplementary file 3 [file EMI4-18-e70433-s005.pdf]

File S1. R Script to assign taxa into categories as non-pathogenic, opportunistic pathogen or pathogen.

```
#!/usr/bin/env Rscript
# ==============================================================================
# classify_bacteria.R   (supplementary material)
# ------------------------------------------------------------------------------
# Reproducible pipeline that assigns each bacterial species a PATHOGENICITY
# class using only facts retrieved from authoritative databases. No LLM
# judgement is used; every class is the output of an explicit, auditable rule
# applied to retrieved evidence, and every reference is copied verbatim from
# the source record.
#
# PRIMARY CLASSIFICATION (pathogenicity only):
#   Pathogen       - primary/frank pathogen (disease in healthy hosts)
#   Opportunist    - commensal/environmental organism that causes disease when
#                    it crosses anatomical barriers or the host is compromised
#                    (documented clinical specimen, BSL >= 2, or clinical
#                    priority-list membership). Opportunist_type = 'rare' flags
a
#                    call resting on a single clinical isolate.
#   Non-pathogenic - no documented disease association
#   Review / blank - insufficient evidence / not inferred (never guessed)
#
# Ecological niche, isolation source, host target, and other potentials
# (priority / animal / plant pathogen, probiotic) are reported in SEPARATE
# descriptive columns so nothing is hidden and the table can be filtered.
#
# Rows the automated criteria cannot classify (Review, or unresolved/blank) are
# flagged in the Needs_curation column for human review; no manual answers are
# embedded in the pipeline.
#
# DEPENDENCIES: base R (libcurl-capable) + jsonlite.  Output: CSV.
# USAGE: Rscript classify_bacteria.R key_species.txt out.csv [limit]
# COMPANION FILES (place beside this script; each optional except the input):
#   key_species.txt   - input species list (required)
#   md2_traits.csv    - MicrobeDirectory 2.0 reference traits (optional)
#   seqcode_names.tsv - SeqCode registry snapshot for name checks (optional)
# ==============================================================================

suppressMessages(library(jsonlite))
options(timeout = 90)
`%||%` <- function(a, b) if (is.null(a) || length(a) == 0 || (length(a) == 1 &&
is.na(a))) b else a
nz <- function(x) !is.null(x) && length(x) == 1 && !is.na(x) &&
nzchar(as.character(x))
NCBI    <- "https://eutils.ncbi.nlm.nih.gov/entrez/eutils"
BACDIVE <- "https://api.bacdive.dsmz.de"
LPSN    <- "https://lpsn.dsmz.de/species"
PAUSE   <- 0.4
CACHE   <- "cache"; dir.create(CACHE, showWarnings = FALSE)

# ---- configurable authority lists (extend for full run) ---------------------
```

```
FRANK_HUMAN <- c("Streptococcus pneumoniae","Streptococcus pyogenes",
  "Haemophilus influenzae","Neisseria gonorrhoeae","Neisseria meningitidis",
  "Corynebacterium diphtheriae","Bordetella pertussis","Salmonella enterica",
  "Vibrio cholerae","Moraxella catarrhalis","Streptococcus agalactiae")
FRANK_HUMAN_GENERA <- c("Mycobacterium","Shigella","Brucella","Treponema",
  "Leptospira","Rickettsia","Chlamydia")
FRANK_PLANT <- c("Xanthomonas oryzae","Clavibacter insidiosus","Agrobacterium
rubi",
  "Pseudomonas syringae","Erwinia amylovora")
FRANK_ANIMAL <- c("Brachyspira hampsonii","Staphylococcus pseudintermedius",
  "Corynebacterium auriscanis","Aquirickettsiella isopodorum")
# Opportunistic organisms of clinical priority (used only as an infection
signal).
PRIORITY_OPP <- c("Acinetobacter baumannii","Pseudomonas aeruginosa",
  "Klebsiella pneumoniae","Staphylococcus aureus","Enterococcus faecium",
  "Enterococcus faecalis","Escherichia coli","Enterobacter hormaechei",
  "Stenotrophomonas maltophilia","Aeromonas caviae","Klebsiella grimontii")
# Verified WHO Bacterial Priority Pathogens List 2024 (species + genus level).
# Source: WHO BPPL 2024, https://www.who.int/publications/i/item/9789240093461
WHO_BPPL_2024 <- c("Acinetobacter baumannii","Klebsiella
pneumoniae","Escherichia coli",
  "Enterobacter hormaechei","Pseudomonas aeruginosa","Staphylococcus aureus",
  "Enterococcus faecium","Neisseria gonorrhoeae","Salmonella enterica",
  "Streptococcus pneumoniae","Haemophilus influenzae","Streptococcus pyogenes",
  "Streptococcus agalactiae")
WHO_BPPL_GENERA <- c("Shigella","Mycobacterium")
PROBIOTIC_GENERA <-
c("Lactobacillus","Lacticaseibacillus","Limosilactobacillus",

"Lactiplantibacillus","Latilactobacillus","Ligilactobacillus","Bifidobacterium",
  "Leuconostoc","Pediococcus")
# Species with established, documented use as probiotics (flagged "probiotic").
ESTABLISHED_PROBIOTICS <- c("Lactobacillus acidophilus","Lacticaseibacillus
rhamnosus",
  "Lacticaseibacillus casei","Lactobacillus helveticus","Lactobacillus
delbrueckii",
  "Lactobacillus crispatus","Bifidobacterium animalis","Bifidobacterium
bifidum",
  "Bifidobacterium longum","Bifidobacterium breve","Lactococcus lactis",
  "Streptococcus thermophilus","Bacillus coagulans","Akkermansia muciniphila",
  "Faecalibacterium prausnitzii")

# ---- source registry: name -> URL (for the per-row column and the footnotes)
--
SOURCES <- c(
  "NCBI Taxonomy"            = "https://www.ncbi.nlm.nih.gov/taxonomy",
  "BacDive"                  = "https://bacdive.dsmz.de",
  "LPSN"                     = "https://lpsn.dsmz.de",
  "WHO BPPL 2024"            =
"https://www.who.int/publications/i/item/9789240093461",
  "EPPO Global Database"     = "https://gd.eppo.int",
  "WOAH listed diseases"     =
"https://www.woah.org/en/what-we-do/animal-health-and-welfare/animal-diseases/",
  "NCBI Pathogen Detection"  = "https://www.ncbi.nlm.nih.gov/pathogens/",
```

```
  "EMBL-EBI Pathogens Portal"= "https://www.pathogensportal.org",
  "SeqCode Registry"         = "https://registry.seqco.de",
  "MD2 MicrobeDirectory 2.0 (reference only, not used in classification)" =
"https://github.com/dcdanko/MD2")
SOURCE_ACCESS <- "accessed 2026-07"

# ---- glossary of terms (written into the table footnotes) ------------------
GLOSSARY <- c(
 "Classification (pathogenicity only):",
 "  Pathogen = primary/frank pathogen; causes disease in healthy hosts (human,
animal or plant).",
 "  Opportunist = commensal/environmental organism that causes disease when it
crosses anatomical",
 "     barriers or the host is immunocompromised.",
 "  Non-pathogenic = no documented disease association.",
 "  Review = evidence ambiguous/conflicting.   (blank) = not inferred; see
Nomenclature.",
 "Opportunist_type: 'rare' = the Opportunist call rests on a single clinical
specimen with biosafety",
 "  level 1 and no priority-list hit (a lone clinical isolate in an
otherwise-commensal/environmental",
 "  organism); blank = a regular Opportunist (>=2 clinical specimens, or BSL>=2,
or priority-listed).",
 "Confidence: high = priority-list hit, BSL>=2, or >=2 clinical specimens;
medium = a single",
 "  clinical specimen (manual review recommended; these are the 'rare'
opportunists).",
 "Classification_evidence: the specific basis for the call - the clinical
specimen(s), BSL, or",
 "  pathogen-list membership behind an Opportunistic/Pathogen call, or why a
Non-pathogenic call was",
 "  made. NOTE: BacDive's bare #Infection category tag is NOT used on its own
(it is routinely",
 "  attached to normal faecal/gut isolates); a genuine clinical specimen is
required.",
 "Pathogen_host_target: intended host of the pathogenicity (Human / Animal /
Plant).",
 "Primary_niche: the single most frequent isolation origin across BacDive
records, one of",
 "  Human / Animal / Food / Environment / Plant (ties broken in that priority
order). Infection/",
 "  clinical records are not counted as a niche.",
 "Primary_isolation_source: the most frequent individual isolation-source string
(ties broken by",
 "  first occurrence). Secondary_isolation_sources: the remaining de-duplicated
sources.",
 "BacDive_source_categories: BacDive GOLD ecosystem paths, Cat1 > Cat2 > Cat3.
Cat1 = Host (from a",
 "  host/host material), Environmental (soil/water/air), Engineered (human-made:
food & fermentation,",
 "  wastewater, built, industry), Infection (clinical specimen). Niche_counts:
votes per niche.",
 "Risk_group_BSL: BacDive biosafety level (1 = no/low risk; 2+ = can cause
disease).",
```

```
 "Other_potentials: WHO/AMR priority (on WHO BPPL 2024); animal pathogen; plant
pathogen;",
 "  probiotic = established documented use; probiotic potential =
probiotic-associated genus only.",
 "Nomenclature: name-resolution notes - LPSN synonym mapping (input -> correct
name), or, for names",
 "  absent from NCBI/LPSN/BacDive, whether the name is registered in the SeqCode
registry snapshot.",
 "Reference / Reference_DOI: original valid-publication citation from LPSN.",
 "Source_databases: every database that contributed evidence to that row.",
 "BacDive_strain_IDs: the BacDive strain record(s) consulted - open any at",
 "  https://bacdive.dsmz.de/strain/<ID>. Clinical specimens in
Classification_evidence name the exact",
 "  strain they came from (e.g. 'Human blood,central venous catheter (BacDive
strain 150566)').",
 "MD2 (MicrobeDirectory 2.0) machine-predicted traits are provided in a SEPARATE
file",
 "  (<output>_md2_reference.csv), reference-only and NOT used in the
classification.",
 "A curation copy (<output>_for_curation.csv) collects all low/medium-confidence
and blank/unresolved",
 "  rows for manual review.",
 "Needs_curation: 'yes' marks rows the automated criteria could not classify
(Review = no/invalid",
 "  BacDive evidence; blank = unresolved in NCBI/LPSN/BacDive), which therefore
require human review.",
 "Rules: R1 frank-pathogen list; R2 opportunistic (clinical specimen / BSL>=2 /
priority list);",
 "  R3 non-pathogenic; R3b BacDive #Infection tag present but only on
non-clinical (faecal/gut)",
 "  samples - discounted; R3c single clinical isolate in a beneficial /
non-clinical-niche (Food/",
 "  Environment/Plant/Animal or probiotic) organism - not treated as
opportunist; R4 no BacDive",
 "  evidence; R0/R5 invalid name / insufficient evidence; MC expert manual
curation (see citation).")

# ---- de-duplicate near-identical isolation-source strings -------------------
# Merges case/spelling variants (Feces/faeces, Yoghurt/yogurt) and BacDive's
# "Category, Sub, detail" duplicates (e.g. "Animal, Rat, feces" == "rat feces").
.samp_cats <- c("animal","environmental","environment","food",
                "plant","other","soil","water","engineered","mammals","birds")
norm_key <- function(s) {
  s <- gsub("<[^>]+>", " ", s); s <- tolower(s)
  s <- gsub("faeces","feces",s); s <- gsub("faecal","fecal",s)
  s <- gsub("caecal","cecal",s); s <- gsub("caecum","cecum",s); s <-
gsub("yoghurt","yogurt",s)
  parts <- trimws(strsplit(s, ",")[[1]])
  while (length(parts) > 1 && parts[1] %in% .samp_cats) parts <- parts[-1]
  s <- paste(parts, collapse = " ")
  trimws(gsub("\\s+", " ", gsub("[^a-z0-9]+", " ", s)))
}
dedup_samples <- function(samples) {
  clean <- trimws(gsub("\\s+", " ", gsub("<[^>]+>", " ", samples)))
```

```
  clean <- clean[nzchar(clean)]
  if (!length(clean)) return(character(0))
  clean[!duplicated(vapply(clean, norm_key, character(1)))]
}


# ---- MD2 reference table (OPTIONAL): attached as reference columns only.
------
# Never used by classify(). Place md2_traits.csv beside this script to enable.
# Joined by NCBI TaxID (primary) with scientific-name fallback.
MD2_BY_TAXID <- new.env(); MD2_BY_NAME <- new.env(); MD2_LOADED <- FALSE
if (file.exists("md2_traits.csv")) {
  md2 <- tryCatch(read.csv("md2_traits.csv", stringsAsFactors = FALSE,
check.names = FALSE),
                  error = function(e) NULL)
  if (!is.null(md2)) {
    MD2_LOADED <- TRUE
    for (i in seq_len(nrow(md2))) {
      rec <- as.list(md2[i, ])
      tid <- sub("\\.0$", "", as.character(rec$taxonomic_id))
      if (!is.na(tid) && nzchar(tid)) assign(tid, rec, envir = MD2_BY_TAXID)
      nm <- tolower(trimws(as.character(rec$scientific_name)))
      if (nzchar(nm)) assign(nm, rec, envir = MD2_BY_NAME)
    }
  }
}
md2_val <- function(rec, field) {
  v <- rec[[field]]
  if (is.null(v) || length(v) == 0 || is.na(v)) return("")
  sub("\\.0$", "", trimws(as.character(v)))
}

# ---- SeqCode registry snapshot (OPTIONAL): used ONLY to annotate names that -
# are unresolved in NCBI/LPSN/BacDive. The registry is comprehensive (it also
# lists ICNP names), so membership is reported as "registered in the SeqCode
# registry", not as proof a name is SeqCode-governed. Bundle seqcode_names.tsv
# (id<TAB>name) beside this script; snapshot date recorded in the footnote.
SEQCODE <- new.env(); SEQCODE_LOADED <- FALSE; SEQCODE_DATE <- "accessed
2026-07"
if (file.exists("seqcode_names.tsv")) {
  sc <- tryCatch(read.delim("seqcode_names.tsv", stringsAsFactors = FALSE, quote
= ""),
                 error = function(e) NULL)
  if (!is.null(sc) && all(c("id","name") %in% names(sc))) {
    SEQCODE_LOADED <- TRUE
    for (i in seq_len(nrow(sc))) assign(tolower(trimws(sc$name[i])),
as.character(sc$id[i]), envir = SEQCODE)
  }
}
seqcode_lookup <- function(sci) {          # returns registry id or "" if absent
  if (!SEQCODE_LOADED) return("")
  k <- tolower(trimws(sci)); if (exists(k, envir = SEQCODE, inherits = FALSE))
get(k, envir = SEQCODE) else ""
}
```

```r
md2_lookup <- function(taxid, sci, resolved) {
  fields <- c("human_pathogen","animal_pathogen","plant_pathogen",
              "pathogenicity","human_microbiome_body_site","gram_stain")
  blank <- setNames(as.list(rep("", length(fields) + 1)), c(paste0("MD2_",
fields), "MD2_matched"))
  if (!MD2_LOADED) return(blank)
  rec <- NULL
  tid <- if (nz(taxid)) sub("\\.0$", "", as.character(taxid)) else ""
  if (nzchar(tid) && exists(tid, envir = MD2_BY_TAXID, inherits = FALSE))
    rec <- get(tid, envir = MD2_BY_TAXID)
  if (is.null(rec)) for (nm in c(resolved, sci)) {
    key <- tolower(trimws(as.character(nm %||% "")))
    if (nzchar(key) && exists(key, envir = MD2_BY_NAME, inherits = FALSE)) { rec
<- get(key, envir = MD2_BY_NAME); break }
  }
  if (is.null(rec)) return(blank)
  out <- setNames(lapply(fields, function(f) md2_val(rec, f)), paste0("MD2_",
fields))
  out[["MD2_matched"]] <- if (!is.null(rec$taxonomic_id)) "by TaxID/name" else
"by name"
  out
}

# ---- http helper (base R, libcurl) with retry -------------------------------
get_text <- function(url, tries = 3) {
  for (i in seq_len(tries)) {
    out <- tryCatch({
      con <- url(url, open = "r")
      on.exit(close(con), add = TRUE)
      paste(suppressWarnings(readLines(con, warn = FALSE)), collapse = "\n")
    }, error = function(e) NULL)
    if (!is.null(out)) return(out)
    Sys.sleep(1.5 * i)
  }
  NULL
}
cache_file <- function(name) file.path(CACHE, name)
slug <- function(s) gsub("[^A-Za-z0-9]+", "_", s)

# ---- NCBI Taxonomy ----------------------------------------------------------
ncbi_resolve <- function(sci) {
  cf <- cache_file(paste0("ncbi_", slug(sci), ".json"))
  if (file.exists(cf)) {
    r <- fromJSON(paste(readLines(cf, warn = FALSE), collapse = ""))
    # Trust the cache only if the previous lookup COMPLETED: it has a TaxID, or
it
    # was flagged ok (a genuine "no match" from a working NCBI query). A blank
left
    # by a transient failure is treated as a miss, so it is retried on the next
run.
    cached_ok <- isTRUE(r$ok) || (!is.null(r$taxid) && !is.na(r$taxid) &&
nzchar(as.character(r$taxid)))
    if (cached_ok)
      return(list(taxid = r$taxid %||% NA, resolved_name = r$resolved_name %||%
```

```
NA, lineage = r$lineage %||% NA))
  }
  q <- URLencode(sci, reserved = TRUE)
  s <-
tryCatch(fromJSON(get_text(sprintf("%s/esearch.fcgi?db=taxonomy&term=%s&retmode=
json", NCBI, q))),
                error = function(e) NULL); Sys.sleep(PAUSE)
  out <- list(taxid = NA, resolved_name = NA, lineage = NA)
  ids <- tryCatch(s$esearchresult$idlist, error = function(e) character(0))
  if (length(ids) > 0) {
    taxid <- ids[1]
    x <- get_text(sprintf("%s/efetch.fcgi?db=taxonomy&id=%s&retmode=xml", NCBI,
taxid)); Sys.sleep(PAUSE)
    out$taxid <- taxid
    if (!is.null(x)) {
      m <- regmatches(x, regexpr("<ScientificName>(.*?)</ScientificName>", x))
      if (length(m)) out$resolved_name <- gsub("</?ScientificName>", "", m)
      l <- regmatches(x, regexpr("<Lineage>(.*?)</Lineage>", x))
      if (length(l)) out$lineage <- gsub("</?Lineage>", "", l)
    }
  }
  # Cache ONLY a positive resolve (a real TaxID + name). Empty results are never
  # cached - whether the name is truly absent from NCBI or the query was
throttled,
  # it is left uncached so the next run retries it (self-healing).
  out$ok <- !is.na(out$resolved_name)
  if (out$ok) writeLines(toJSON(out, auto_unbox = TRUE), cf)
  out
}

# ---- helper: coerce "single object OR list of objects" to a list -----------
as_entry_list <- function(x) {
  if (is.null(x)) return(list())
  if (is.list(x) && (!is.null(x[["Cat1"]]) || !is.null(x[["sample type"]]) ||
!is.null(x[["biosafety level"]])))
    return(list(x))
  if (!is.list(x)) return(list())
  Filter(is.list, x)
}

# ---- map a BacDive source category (Cat1/Cat2/Cat3) to one of five niches ----
# Human / Animal / Food / Environment / Plant  (Infection/Condition are not
niches)
niche_of_cat <- function(c1, c2, c3, has_human) {
  cc <- tolower(paste(c1, c2, c3))
  if (grepl("food|fermented|dairy|beverage|cheese|yogurt|yoghurt|milk product",
cc)) return("Food")
  if
(grepl("plant|phyllosphere|leaf|rhizosph|root|flower|seed|fruit|pollen|phytopath
", cc)) return("Plant")
  if (startsWith(c1, "Host")) return(if (has_human) "Human" else "Animal")
  if (c1 == "Environmental") return("Environment")
  if (c1 == "Engineered")   return("Environment")   # human-made, non-food
(wastewater, built, industrial)
```

```
  if (c1 %in% c("Infection", "Condition")) return(NA_character_)
  NA_character_
}

# ---- BacDive ------------------------------------------------------------
bacdive_evidence <- function(genus, species) {
  cf <- cache_file(paste0("bacdive_", genus, "_", species, ".json"))
  if (file.exists(cf)) return(fromJSON(readLines(cf, warn = FALSE),
simplifyVector = FALSE))
  idx <- tryCatch(fromJSON(get_text(sprintf("%s/taxon/%s/%s", BACDIVE, genus,
species))),
                  error = function(e) NULL); Sys.sleep(PAUSE)
  ids <- tryCatch(idx$results, error = function(e) NULL)
  entries <- list(); samples <- character(0); bsls <- character(0); sample_src
<- list()
  used_ids <- character(0)
  if (length(ids) > 0) for (bid in head(ids, 5)) {
    rec <- tryCatch(fromJSON(get_text(sprintf("%s/fetch/%s", BACDIVE, bid)),
simplifyVector = FALSE),
                    error = function(e) NULL); Sys.sleep(PAUSE)
    if (is.null(rec)) next
    res <- if (!is.null(rec$results)) rec$results else rec
    if (length(res) == 0) next          # empty/malformed record - skip safely
    used_ids <- c(used_ids, as.character(bid))
    r0 <- res[[1]]
    iso <- r0[["Isolation, sampling and environmental information"]]
    for (it in as_entry_list(iso[["isolation"]]))
      if (!is.null(it[["sample type"]])) {
        samples <- c(samples, it[["sample type"]])
        sample_src[[length(sample_src)+1]] <- list(bid = as.character(bid), s =
it[["sample type"]])
      }
    for (it in as_entry_list(iso[["isolation source categories"]]))
      if (!is.null(it[["Cat1"]]))
        entries[[length(entries)+1]] <- list(c1 = sub("^#","",it[["Cat1"]]),
                                             c2 =
sub("^#","",ifelse(is.null(it[["Cat2"]]),"",it[["Cat2"]])),
                                             c3 =
sub("^#","",ifelse(is.null(it[["Cat3"]]),"",it[["Cat3"]])),
                                             bid = as.character(bid))
    for (it in as_entry_list(r0[["Safety information"]][["risk assessment"]]))
      if (!is.null(it[["biosafety level"]])) bsls <- c(bsls,
as.character(it[["biosafety level"]]))
  }
  samples <- dedup_samples(unique(samples)); bsls <- unique(bsls)
  stxt <- tolower(paste(samples, collapse = " "))
  CLIN_RE <-
"\\binfection\\b|infected|abscess|\\bwound\\b|sepsis|septic|bacter[a]?emia|\\bpu
s\\b|empyema|peritonitis|endocarditis|meningitis|osteomyelitis|\\bblood\\b|cereb
rospinal|\\bcsf\\b|pleural|synovial|\\bclinical\\b|catheter|cornea|keratitis|con
junctiv|ocular|otitis|sputum|bronchoalveolar|ventilator|urinary
tract|\\blesion\\b|biopsy|aspirate|nosocomial|prosthe"
  has_human <- any(vapply(entries, function(e) e$c2 == "Human", logical(1))) ||
    grepl("human|patient|infant|homo sapiens|neonat", stxt)
```

```
  b <- c(human = 0, animal = 0, environmental = 0, infection = 0, other = 0)
  for (e in entries) {
    c1 <- e$c1
    if (startsWith(c1, "Host")) {
      if (has_human) b["human"] <- b["human"] + 1 else b["animal"] <-
b["animal"] + 1
    } else if (c1 %in% c("Environmental", "Engineered")) {
      b["environmental"] <- b["environmental"] + 1
    } else if (c1 %in% c("Infection", "Condition")) {
      b["infection"] <- b["infection"] + 1
    } else {
      b["other"] <- b["other"] + 1
    }
  }
  cat1 <- unique(vapply(entries, function(e) e$c1, character(1)))
  clin_samples <- samples[grepl(CLIN_RE, tolower(samples))]  # actual
clinical/infection specimens
  clinical <- length(clin_samples) > 0
  healthy  <- grepl("healthy|normal flora", stxt)
  # link each clinical specimen to the BacDive strain it came from (provenance)
  clin_refs <- character(0)
  for (ss in sample_src) if (grepl(CLIN_RE, tolower(ss$s)))
    clin_refs <- c(clin_refs, sprintf("%s (BacDive strain %s)",
trimws(gsub("<[^>]+>"," ",ss$s)), ss$bid))
  clin_refs <- unique(clin_refs)

  # --- five-category niche tally (one vote per source-category entry) ---------
  niche_votes <- character(0)
  for (e in entries) {
    nv <- niche_of_cat(e$c1, e$c2, e$c3, has_human)
    if (!is.na(nv)) niche_votes <- c(niche_votes, nv)
  }
  NICHE_PRIORITY <- c("Human","Animal","Food","Plant","Environment")   #
tie-break order
  primary_niche <- ""; niche_counts_str <- ""
  if (length(niche_votes)) {
    tb <- table(niche_votes)
    ord <- order(-as.integer(tb), match(names(tb), NICHE_PRIORITY))
    primary_niche <- names(tb)[ord[1]]
    niche_counts_str <- paste(sprintf("%s:%d", names(tb)[ord],
as.integer(tb)[ord]), collapse = "; ")
  }
  # --- primary vs secondary isolation source (most frequent source string) ----
  norm_freq <- table(vapply(samples, norm_key, character(1)))
  primary_src <- ""; secondary_src <- character(0)
  if (length(samples)) {
    keys <- vapply(samples, norm_key, character(1))
    ordS <- order(-as.integer(norm_freq[keys]), seq_along(samples))   # most
frequent, then first-seen
    primary_src <- samples[ordS[1]]
    secondary_src <- samples[ordS[-1]]
  }
  # --- BacDive Cat1 > Cat2 > Cat3 paths ---------------------------------------
  paths <- unique(vapply(entries, function(e)
```

```
    paste(Filter(nzchar, c(e$c1, e$c2, e$c3)), collapse = " > "), character(1)))

  out <- list(n_records = length(ids), cat1 = cat1, buckets = as.list(b),
              primary_niche = primary_niche, niche_counts = niche_counts_str,
              primary_source = trimws(gsub("<[^>]+>"," ", primary_src)),
              secondary_sources = secondary_src,
              source_categories = paths,
              bsl = bsls, buckets = as.list(b), has_human = has_human,
              clinical = clinical, n_clinical = length(clin_samples),
              clinical_samples = clin_samples, clinical_refs = clin_refs,
              bacdive_ids = unique(used_ids), healthy = healthy)
  writeLines(toJSON(out, auto_unbox = TRUE), cf); out
}

# ---- LPSN reference ----------------------------------------------------------
lpsn_reference <- function(genus, species) {
  cf <- cache_file(paste0("lpsn_", genus, "_", species, ".json"))
  if (file.exists(cf)) {
    r <- fromJSON(paste(readLines(cf, warn = FALSE), collapse = ""))
    return(list(reference = r$reference %||% NA, doi = r$doi %||% NA,
                status = r$status %||% NA, correct_name = r$correct_name %||%
NA))
  }
  page <- get_text(sprintf("%s/%s-%s", LPSN, tolower(genus), tolower(species)));
Sys.sleep(PAUSE)
  out <- list(reference = NA, doi = NA, status = NA, correct_name = NA)
  if (!is.null(page)) {
    m <- regmatches(page, regexpr("(?s)(?:Valid|Effective)
publication:\\s*(.*?)(?:<a |</p>|<br)", page, perl = TRUE))
    if (length(m)) {
      ref <- sub("(?s)(?:Valid|Effective) publication:", "", m, perl = TRUE)
      ref <- gsub("<[^>]*>|<a\\s*$", "", ref)
      out$reference <- trimws(gsub("\\s+", " ", ref))
    }
    d <- regmatches(page, regexpr("https://doi.org/[^\"]+", page, perl = TRUE))
    if (length(d)) out$doi <- d[1]
    s <- regmatches(page, regexpr("correct name|validly published under the
ICNP|synonym", page, perl = TRUE))
    if (length(s)) out$status <- s[1]
    # synonym: LPSN states the correct name as a linked species slug
    cm <- regexec('Correct name:(?:\\s|<[^>]+>)*<a
href="/species/([a-z0-9-]+)"', page, perl = TRUE)
    cg <- regmatches(page, cm)[[1]]
    if (length(cg) >= 2) {
      p <- strsplit(cg[2], "-")[[1]]
      out$correct_name <- paste0(toupper(substr(p[1], 1, 1)), substr(p[1], 2,
nchar(p[1])),
                                 " ", paste(p[-1], collapse = " "))
    }
  }
  writeLines(toJSON(out, auto_unbox = TRUE), cf); out
}

# ---- descriptive niche (infection isolates excluded from host tally) --------
```

```
niche_of <- function(bd) {
  b <- bd$buckets
  num <- function(x) if (is.null(x)) 0 else as.numeric(x)
  h <- num(b$human); a <- num(b$animal); e <- num(b$environmental)
  if (h == 0 && a == 0 && e == 0) return("Unknown")
  if (h > 0 && e > 0 && abs(h - e) <= 1) return("Mixed (host + environmental)")
  if (h > 0 && h >= e && h >= a) return("Host-associated (human)")
  if (e > 0 && e >= h && e >= a) return("Environmental / food / built")
  if (a > 0 && h == 0)           return("Host-associated (animal)")
  "Mixed"
}

# ---- rule-based pathogenicity classifier ----------------------------------
classify <- function(sci, genus, bd) {
  b <- bd$buckets; bsl <- unlist(bd$bsl)
  n_rec <- if (length(bd$n_records)) as.numeric(bd$n_records) else 0
  rg2 <- any(bsl %in% c("2","3","4"))
  infb <- if (is.null(b$infection)) 0 else as.numeric(b$infection)
  had_infection_tag <- (infb > 0)              # BacDive #Infection category tag
present
  frank_h <- sci %in% FRANK_HUMAN || genus %in% FRANK_HUMAN_GENERA
  frank_p <- sci %in% FRANK_PLANT
  frank_a <- sci %in% FRANK_ANIMAL
  priority <- sci %in% PRIORITY_OPP
  has_human <- isTRUE(unlist(bd$has_human))
  clinical  <- isTRUE(unlist(bd$clinical))     # an actual clinical/infection
specimen (blood, catheter, cornea...)
  n_clin    <- if (length(bd$n_clinical)) as.numeric(bd$n_clinical) else 0
  # Opportunistic requires REAL evidence: a clinical specimen, BSL>=2, or a
  # priority-list hit. BacDive's bare #Infection category tag is NOT sufficient
  # (it is routinely attached to normal faecal/gut isolates).
  opportunistic <- clinical || rg2 || priority

  if (frank_h) return(list(cls="Pathogen", target="Human", rule="R1: frank
human-pathogen list", conf="high"))
  if (frank_p) return(list(cls="Pathogen", target="Plant", rule="R1:
plant-pathogen list", conf="high"))
  if (frank_a) return(list(cls="Pathogen", target="Animal", rule="R1:
animal-pathogen list", conf="high"))
  if (opportunistic) {
    tgt <- if (has_human || priority) "Human" else "Animal/other"
    rare <- (n_clin == 1 && !rg2 && !priority)   # lone clinical isolate, BSL 1,
not priority
    conf <- if (priority || rg2 || n_clin >= 2) "high" else "medium"
    return(list(cls="Opportunist", target=tgt, otype = if (rare) "rare" else "",
                rule=paste0("R2: clinical specimen / BSL>=2 / priority-list
evidence",
                            if (rare) " (rare: single clinical specimen, BSL 1,
not priority)" else ""),
                conf=conf))
  }
  if (had_infection_tag)
    return(list(cls="Non-pathogenic", target="",
                rule="R3b: BacDive #Infection tag present but only on
```

```
non-clinical (e.g. faecal) samples - discounted",
                conf="high"))
  if (n_rec == 0) return(list(cls="Review", target="", rule="R4: no BacDive
evidence", conf="low"))
  list(cls="Non-pathogenic", target="", rule="R3: no documented disease
association", conf="high")
}

flags_of <- function(sci, genus, bd) {
  f <- character(0)
  if (sci %in% WHO_BPPL_2024 || genus %in% WHO_BPPL_GENERA) f <- c(f, "WHO/AMR
priority")
  if (sci %in% FRANK_ANIMAL) f <- c(f, "animal pathogen")
  if (sci %in% FRANK_PLANT)  f <- c(f, "plant pathogen")
  if (sci %in% ESTABLISHED_PROBIOTICS) f <- c(f, "probiotic")
  else if (genus %in% PROBIOTIC_GENERA) f <- c(f, "probiotic potential")
  paste(f, collapse = "; ")
}

# ---- plain-language evidence for each classification (auditable column) -----
evidence_of <- function(sci, genus, bd, cl) {
  cls <- cl$cls
  if (identical(cls, "Pathogen")) {
    src <- if (sci %in% FRANK_PLANT) "EPPO plant-pathogen list"
           else if (sci %in% FRANK_ANIMAL) "WOAH / veterinary animal-pathogen
list"
           else "frank human-pathogen list / WHO BPPL 2024"
    return(sprintf("Listed as a primary %s pathogen (%s).", tolower(cl$target),
src))
  }
  if (identical(cls, "Opportunist")) {
    parts <- character(0)
    cs <- unlist(bd$clinical_refs); if (!length(cs)) cs <-
unlist(bd$clinical_samples)
    if (length(cs)) parts <- c(parts, paste0("clinical specimen(s): ",
paste(head(cs, 3), collapse = "; ")))
    bsl <- unlist(bd$bsl); rg <- bsl[bsl %in% c("2","3","4")]
    if (length(rg)) parts <- c(parts, paste0("BacDive biosafety level ",
paste(rg, collapse = "/")))
    if (sci %in% PRIORITY_OPP) parts <- c(parts, "on WHO/AMR priority list")
    if (!length(parts)) parts <- "documented infection association"
    note <- if (identical(cl$otype, "rare")) " Flagged rare (single clinical
specimen, BSL 1, not priority)." else ""
    return(paste0("Opportunist evidence - ", paste(parts, collapse = "; "), ".",
note))
  }
  if (identical(cls, "Non-pathogenic")) {
    if (grepl("R3b", cl$rule))
      return("No clinical isolate. A BacDive #Infection tag is present but only
on non-clinical (e.g. faecal/gut) samples, so it was discounted.")
    return("No clinical/infection specimen, biosafety level 1, and no
pathogen-list membership; normal-flora / environmental sources only.")
  }
  if (identical(cls, "Review")) return(cl$rule)
```

```
  ""
}

# ---- per-row source attribution (every database that contributed evidence) --
source_dbs <- function(sci, genus, bd, lp) {
  s <- "NCBI Taxonomy"
  if (length(bd$n_records) && bd$n_records > 0) s <- c(s, "BacDive")
  if (nz(lp$reference))  s <- c(s, "LPSN")
  if (sci %in% WHO_BPPL_2024 || genus %in% WHO_BPPL_GENERA) s <- c(s, "WHO BPPL 
2024")
  if (sci %in% FRANK_PLANT)  s <- c(s, "EPPO Global Database")
  if (sci %in% FRANK_ANIMAL) s <- c(s, "WOAH listed diseases")
  paste(unique(s), collapse = "; ")
}

# ---- main --------------------------------------------------------------------
# Run from a terminal:  Rscript classify_bacteria.R key_species.txt output.csv 
[limit]
# Or in RStudio: setwd() to this folder, then click Source (defaults are used 
below).
args <- commandArgs(trailingOnly = TRUE)
infile  <- if (length(args) >= 1) args[1] else "key_species.txt"
outfile <- if (length(args) >= 2) args[2] else "classification_output.csv"
lim     <- if (length(args) >= 3) as.integer(args[3]) else 0L
names <- trimws(readLines(infile, warn = FALSE)); names <- names[names != ""]
if (tolower(names[1]) %in% c("taxa","species","genus_species")) names <- 
names[-1]
if (lim > 0) names <- head(names, lim)

cols <- 
c("Input_name","Resolved_name","NCBI_TaxID","Lineage","Classification","Opportun
ist_type",
          
"Pathogen_host_target","Confidence","Rule_applied","Classification_evidence",
          
"Primary_niche","Primary_isolation_source","Secondary_isolation_sources",
          
"BacDive_source_categories","Niche_counts","BacDive_records","Risk_group_BSL",
          
"Other_potentials","Reference","Reference_DOI","LPSN_status","Nomenclature",
          "Source_databases","BacDive_strain_IDs")
res <- data.frame(matrix(ncol = length(cols), nrow = 0), stringsAsFactors = 
FALSE); names(res) <- cols

# MD2 machine-predicted traits are written to a SEPARATE reference file (only
# for species that actually match), never mixed into the classification table.
md2_cols <- 
c("Input_name","Resolved_name","NCBI_TaxID","MD2_human_pathogen","MD2_animal_pat
hogen",
              
"MD2_plant_pathogen","MD2_pathogenicity","MD2_gram_stain","MD2_human_microbiome_
body_site")
md2_res <- data.frame(matrix(ncol = length(md2_cols), nrow = 0), 
stringsAsFactors = FALSE); names(md2_res) <- md2_cols
```

```
# discovered LPSN synonyms are written to this map (created/updated as we go)
SYN_FILE <- "synonyms_lpsn.csv"
record_synonym <- function(old, new) {
  if (!file.exists(SYN_FILE)) writeLines("input_name,lpsn_correct_name",
SYN_FILE)
  existing <- readLines(SYN_FILE, warn = FALSE)
  if (!any(grepl(paste0("^\"?", old), existing)))
    cat(sprintf('"%s","%s"\n', old, new), file = SYN_FILE, append = TRUE)
}

for (i in seq_along(names)) {
  raw <- names[i]; sci <- trimws(gsub("_", " ", raw))
  parts <- strsplit(sci, " ")[[1]]; genus <- parts[1]
  species <- if (length(parts) > 1) parts[2] else ""
  malformed <- species %in% c("", "spp", "spp.", "sp", "sp.")
  nomen <- ""; unresolved <- FALSE
  if (malformed) {
    nc <- ncbi_resolve(sci)
    bd <- list(n_records = 0, cat1 = character(0), bsl = character(0),
               primary_niche = "n/a", niche_counts = "", primary_source = "",
               secondary_sources = character(0), source_categories =
character(0),
               has_human = FALSE, bacdive_ids = character(0),
               clinical = FALSE, n_clinical = 0, clinical_samples =
character(0), clinical_refs = character(0))
    lp <- list(reference = NA, doi = NA, status = NA, correct_name = NA)
    cl <- list(cls="Review", target="", rule="R0: not a valid species binomial",
conf="low")
    nomen <- "not a valid species binomial"
  } else {
    lp <- lpsn_reference(genus, species)
    nc <- ncbi_resolve(sci)                     # try the name AS PROVIDED
first
    # LPSN sometimes flags a name as a synonym of another. Only rewrite to
LPSN's
    # correct name when the provided name does NOT resolve in NCBI (an obsolete
input
    # name, e.g. Eubacterium rectale -> Agathobacter rectalis). If the provided
name
    # IS current in NCBI but LPSN disagrees (e.g. Mammaliicoccus sciuri, which
LPSN
    # treats as Staphylococcus sciuri), keep the provided name and just record
the note.
    if (nz(lp$correct_name) && tolower(lp$correct_name) != tolower(sci)) {
      if (is.na(nc$taxid %||% NA)) {
        nomen <- sprintf("LPSN synonym: input '%s' -> correct name '%s'
(adopted; input not current in NCBI)", sci, lp$correct_name)
        record_synonym(sci, lp$correct_name)
        sci <- lp$correct_name
        parts <- strsplit(sci, " ")[[1]]; genus <- parts[1]
        species <- if (length(parts) > 1) parts[2] else ""
        lp2 <- lpsn_reference(genus, species); if (nz(lp2$reference) ||
nz(lp2$correct_name)) lp <- lp2
```

```
        nc <- ncbi_resolve(sci)
      } else {
        nomen <- sprintf("LPSN regards '%s' as a synonym of '%s'; kept as
provided (current name in NCBI).", sci, lp$correct_name)
      }
    }
    bd <- bacdive_evidence(genus, species)
    unresolved <- is.na(nc$taxid %||% NA) && (!length(bd$n_records) ||
bd$n_records == 0) && is.na(lp$reference %||% NA)
    if (unresolved) {
      cl <- list(cls="", target="", rule="Not inferred: no NCBI / LPSN / BacDive
record", conf="")
      sid <- seqcode_lookup(sci)
      if (nzchar(sid))
        nomen <- sprintf("Not in NCBI/LPSN/BacDive; registered in the SeqCode
registry (registry.seqco.de/names/%s, %s). Classification not inferred.", sid,
SEQCODE_DATE)
      else if (!nzchar(nomen))
        nomen <- "Unresolved name - not found in NCBI/LPSN/BacDive or the
SeqCode registry snapshot. Classification not inferred."
    } else {
      cl <- classify(sci, genus, bd)
    }
  }
  srcs <- if (unresolved) "" else source_dbs(sci, genus, bd, lp)
  ev <- if (unresolved) "" else evidence_of(sci, genus, bd, cl)
  # --- R3c: rare-Opportunist exception
-----------------------------------------
  # A documented beneficial/probiotic organism, or one whose primary niche is
  # non-clinical (Food/Environment/Plant/Animal), is NOT labelled an opportunist
on
  # the strength of a single clinical isolate (the 'rare' tier). The isolate is
kept
  # in the evidence but the call is downgraded to Non-pathogenic.
  if (identical(cl$cls, "Opportunist") && identical(cl$otype %||% "", "rare")) {
    pn <- bd$primary_niche %||% ""
    if (pn %in% c("Food","Environment","Plant","Animal") || grepl("probiotic",
flags_of(sci, genus, bd))) {
      ev <- sprintf("Reclassified from rare Opportunist (R3c): only clinical
evidence is a single isolate (%s); primary niche '%s' / beneficial, so not
treated as an opportunist.",
                    paste(head(unlist(bd$clinical_refs), 2), collapse="; "), if
(nzchar(pn)) pn else "non-clinical")
      cl$cls <- "Non-pathogenic"; cl$otype <- ""; cl$conf <- "high"
      cl$rule <- "R3c: single clinical isolate in a beneficial /
non-clinical-niche organism - not treated as opportunist"
    }
  }
  md2 <- md2_lookup(nc$taxid, sci, nc$resolved_name)
  res[nrow(res)+1, ] <- list(raw, nc$resolved_name %||% "", nc$taxid %||% "",
nc$lineage %||% "",
    cl$cls, cl$otype %||% "", cl$target, cl$conf, cl$rule, ev,
    bd$primary_niche %||% "", bd$primary_source %||% "",
    paste(unlist(bd$secondary_sources), collapse=" | "),
```

```
    paste(unlist(bd$source_categories), collapse=" | "), bd$niche_counts %||%
"",
    bd$n_records, paste(bd$bsl, collapse=", "),
    flags_of(sci, genus, bd), lp$reference %||% "", lp$doi %||% "", lp$status
%||% "", nomen, srcs,
    paste(unlist(bd$bacdive_ids), collapse="; "))
  if (nzchar(md2$MD2_matched))
    md2_res[nrow(md2_res)+1, ] <- list(raw, nc$resolved_name %||% "", nc$taxid
%||% "",
      md2$MD2_human_pathogen, md2$MD2_animal_pathogen, md2$MD2_plant_pathogen,
      md2$MD2_pathogenicity, md2$MD2_gram_stain,
md2$MD2_human_microbiome_body_site)
  cat(sprintf("[%d/%d] %-38s -> %s (%s)\n", i, length(names), sci,
              if (nzchar(cl$cls)) cl$cls else "(not inferred)", cl$conf))
}
`%||%` <- function(a, b) if (is.null(a) || length(a) == 0 || is.na(a)) b else a
# ---- highlight rows the automated criteria could not classify ---------------
# Any row left as Review (no / invalid BacDive evidence) or blank (unresolved in
# NCBI/LPSN/BacDive) cannot be assigned by the logical criteria and is flagged
# for human review. No manual answers are embedded in the pipeline.
res$Needs_curation <- ifelse(res$Classification %in% c("Review", ""), "yes", "")
write.csv(res, outfile, row.names = FALSE, na = "")
# ---- append glossary + source footnotes to the bottom of the table ----------
fn <- file(outfile, "a")
writeLines("", fn)
writeLines("# ====================  GLOSSARY OF TERMS  ====================",
fn)
for (g in GLOSSARY) writeLines(paste0("# ", g), fn)
writeLines("#", fn)
writeLines(sprintf("# ====================  DATA SOURCES (%s)
====================", SOURCE_ACCESS), fn)
for (nm in names(SOURCES)) writeLines(sprintf("# [%s] %s", nm, SOURCES[[nm]]),
fn)
close(fn)
cat(sprintf("\nWrote %d rows (+ glossary & source footnotes) to %s\n",
nrow(res), outfile))

# ---- separate MD2 reference file (only species with MD2 data) ---------------
md2file <- sub("\\.csv$", "_md2_reference.csv", outfile)
write.csv(md2_res, md2file, row.names = FALSE, na = "")
cat(sprintf("Wrote %d MD2 reference rows to %s\n", nrow(md2_res), md2file))

# ---- curation copy: low/medium confidence + blank/unresolved rows -----------
curfile <- sub("\\.csv$", "_for_curation.csv", outfile)
cur <- res[res$Confidence %in% c("low","medium") | res$Classification == "", ,
drop = FALSE]
write.csv(cur, curfile, row.names = FALSE, na = "")
cat(sprintf("Wrote %d rows needing manual curation to %s\n", nrow(cur),
curfile))
```
